# Neurotropic Murine β-Coronavirus Infection Causes Differential Expression of Connexin 47 in Oligodendrocyte Subpopulations Associated with Demyelination

**DOI:** 10.1007/s12035-024-04482-0

**Published:** 2024-09-18

**Authors:** Soubhik Das, Archana Kumari Shaw, Subhajit Das Sarma, Michael Koval, Jayasri Das Sarma, Mahua Maulik

**Affiliations:** 1 Biotechnology Research and Innovation Council - National Institute of Biomedical Genomics (BRIC-NIBMG), Kalyani, 741251 West Bengal India; 2 Department of Biological Sciences, Indian Institute of Science Education and Research Kolkata, Mohanpur, 741246 West Bengal India; 3 Departments of Medicine and Cell Biology, Emory University School of Medicine, Atlanta, GA 30322 USA

**Keywords:** Oligodendrocytes, Connexin 47, Gap junction, Connexin 43, Mouse β-coronavirus, Spinal cord demyelination

## Abstract

Gap junctions (GJs) play a crucial role in the survival of oligodendrocytes and myelination of the central nervous system (CNS). In this study, we investigated the spatiotemporal changes in the expression of oligodendroglial GJ protein connexin 47 (Cx47), its primary astroglial coupling partner, Cx43, and their association with demyelination following intracerebral infection with mouse hepatitis virus (MHV). Neurotropic strains of MHV, a β-coronavirus, induce an acute encephalomyelitis followed by a chronic demyelinating disease that shares similarities with the human disease multiple sclerosis (MS). Our results reveal that Cx47 GJs are persistently lost in mature oligodendrocytes, not only in demyelinating lesions but also in surrounding normal appearing white and gray matter areas, following an initial loss of astroglial Cx43 GJs during acute infection. At later stages after viral clearance, astroglial Cx43 GJs re-emerge but mature oligodendrocytes fail to fully re-establish GJs with astrocytes due to lack of Cx47 GJ expression. In contrast, at this later demyelinating stage, the increased oligodendrocyte precursor cells appear to exhibit Cx47 GJs. Our findings further highlight varying degrees of demyelination in distinct spinal cord regions, with the thoracic cord showing the most pronounced demyelination. The regional difference in demyelination correlates well with dynamic changes in the proportion of different oligodendrocyte lineage cells exhibiting differential Cx47 GJ expression, suggesting an important mechanism of progressive demyelination even after viral clearance.

**Supplementary Information:**

The online version contains supplementary material available at 10.1007/s12035-024-04482-0.

## Introduction

Multiple sclerosis (MS) is a chronic autoimmune demyelinating disease of the central nervous system (CNS) which remains of unknown etiology. It is marked by a substantial merging of primary demyelinating plaques accompanied by varying degrees of axonal degeneration, inflammation, and astrogliosis [1]. The subsequent repair mechanisms aimed at restoring the usual myelin structure, referred to as remyelination, are either incomplete or fail completely, resulting in the development of a chronic progressive form of the disease within a decade to 15 years [2, 3]. Both genetic and environmental factors are believed to play important roles in the disease onset and progression. Particularly, viral infection has long been suggested as a contributing factor in both the initiation and worsening of the disease. However, a precise viral agent responsible for these processes has not been identified to date. One possible explanation for the challenge in pinpointing one or more viral pathogens as the direct cause of subsequent demyelination is that the virus may have been cleared from the system by the time the brains and spinal cords are analyzed.

Murine β-coronaviruses (CoV) including certain strains of mouse hepatitis virus (MHV-A59 and JHM) have been widely used as experimental models to gain insights into the mechanisms of demyelination in MS [4–9]. MHV is genetically linked to the severe acute respiratory syndrome coronavirus (SARS-CoV), the Middle East respiratory syndrome coronavirus (MERS-CoV), and the newly discovered SARS-CoV2, which is responsible for the recent COVID-19 pandemic [10]. Most mice that survive acute MHV infection go on to develop a persistent demyelinating disease. MHV-A59 undergoes replication in the brain, spinal cord, and liver, with replication reaching its peak around 5 days post-infection (pi). Subsequently, between 10 and 14 days pi, the infectious virus is completely cleared from all organs, but viral RNA persists in the CNS. This persistence of viral RNA in the CNS is accompanied by the development of inflammatory demyelination, which reaches its peak in the spinal cord at around 1 month after infection [7, 9, 11–13]. The MHV-induced CNS disease encompasses a pattern of recurring demyelination and spontaneous remyelination in the spinal cords of infected mice [9, 14], indicating that this model shares pathological similarities with the human demyelinating disease MS.

There is now considerable evidence suggesting that maintenance of the panglial gap junction (GJ) connectivity in the CNS is vital for limiting demyelination and promoting repair. GJs are intercellular communicating channels comprising of proteins called connexins (Cxs) that facilitate the transport of ions and small metabolites across extended cellular regions [15–19]. They are made up of two connexons (hemichannels), with each connexon originating from adjacent cells. Each connexon consists of six connexin protein subunits. When identical connexons couple together, they form homotypic GJ channels, while heterotypic channels are created when two different hemichannels dock to each other. Astrocytes mostly express connexin 43 (Cx43) and Cx30, along with Cx26 being expressed in a limited subpopulation of gray matter astrocytes [20]. Oligodendrocytes, on the other hand, express Cx47, Cx32, and Cx29, but it is worth noting that Cx29 does not form functional GJ channels in vitro or in vivo [21–23]. The expression of connexins in both oligodendrocytes and astrocytes leads to functional coupling between astrocytes (A/A), between oligodendrocytes (O/O), and between oligodendrocytes and astrocytes (O/A) [23, 24]. Among the glial connexins, Cx47 and its astroglial counterpart, Cx43, are crucial for preserving CNS myelin, particularly in white matter, due to their predominant role in forming the majority of O/A GJs [25, 26]. Mutations in the gene *GJC2* encoding Cx47 cause Pelizaeus-Merzbacher-like disease, a hypomyelinating leukodystrophy, while astrocytic Cx43 mutations contribute to demyelination in oculodentodigital dysplasia syndrome, underscoring the importance of oligodendrocyte-astrocyte connectivity. Further, studies on several connexin knock-out mice emphasize the importance of O/A coupling for myelin maintenance [25].

Extensive changes in glial Cxs have been documented in various acquired human demyelinating conditions including MS [27–30]. In acute MS plaques, there is a more pronounced reduction in oligodendroglial Cx32 and Cx47 as well as astroglial Cx43 compared to demyelinated regions, despite the continued presence of oligodendroglia and astroglia. In contrast, chronic MS lesions display significant upregulation of astroglial Cx43, indicating ongoing astrogliosis and enduring loss of oligodendroglial Cx32 and Cx47, even in partially remyelinated shadow plaques [27, 28]. Similar changes in glial connexins in the presence of astrocytes and oligodendrocytes have also been reported in active demyelinating lesions of other human demyelinating diseases like Balo’s diseases, a variant of MS, suggesting a contribution of disrupted astrocyte-oligodendrocyte and myelin interaction to the disease pathology [30]. These changes in glial Cxs have also been replicated in the experimental autoimmune encephalomyelitis (EAE) mouse model of the disease [31]. Yet, the impact on this intricately specialized panglial GJ network during a viral-induced demyelination has not been thoroughly investigated. Compared to Cx32, Cx47 is more abundantly expressed on the surface of oligodendroglial cell bodies and the proximal parts of their processes, while showing similar expression levels on the surface of myelin sheaths in both white and gray matter [21, 32]. Cx47 forms homotypic GJs between oligodendroglial cells (O/O) with itself, heterotypic O/O GJs with Cx32, and most heterotypic GJs with Cx43 in oligodendroglia-astroglia (O/A) connections [21, 25, 26]. Previously, it has been shown that Cx47 expression is downregulated in association with demyelination in brain tissue of mice infected with MHV-A59 [33]. However, how the altered Cx47 expression in oligodendrocytes correlates with the expression of its primary astroglial GJ coupling partner, Cx43, and influences the demyelinating pathology induced remains elusive.

The present study thus investigates the temporal and spatial changes in Cx47 GJ expression in association with the alterations of Cx43 and myelin markers in mouse spinal cord tissue, which exhibits more pronounced demyelination compared to brain following intracerebral MHV-A59 infection. Our findings highlight regional variations in demyelination within the spinal cord following infection which correlates well with the altered expression of Cx47 GJs in different oligodendroglial lineage cell populations suggesting a possible mechanistic link with persistent demyelination.

## Material and Methods

### Reagents

Bicinchoninic acid (BCA) protein assay kit; 4–12% NuPAGE Bis–Tris gels; SuperSignal West Pico PLUS enhanced chemiluminescence kit; Alexa Fluor 488, 568, and 647 conjugated secondary antibodies; Tyramide signal amplification kit; FluoroMyelin red fluorescent myelin stain; and ProLong Diamond antifade reagent were all purchased from Thermo Fisher Scientific, Inc. Protease and phosphatase inhibitor cocktails were obtained from Sigma-Aldrich, Inc. UltraFreeze® (OCT) frozen section medium and AutoFrost® charged glass slides were obtained from Cancer Diagnostics, Inc. Sources of all the primary antibodies used in the study are listed in Table 1. All secondary antibodies conjugated with horseradish peroxidase were from Jackson ImmunoResearch Laboratories, Inc. All other chemicals were from Sigma-Aldrich Chemical Pvt. Ltd. or Thermo Fisher Scientific, Inc.

**Table 1:** Details of the primary antibodies used in the study

Antibody	IF dilution	WB dilution	Source
Rabbit polyclonal anti-connexin 47	1:400	1:1000	Thermo Fisher Scientific, Inc
Rabbit polyclonal anti-connexin 43	1:400	1:20,000	Sigma-Aldrich, Inc
Goat polyclonal anti-connexin 43	1:100	n/a	MyBioSource, Inc
Mouse monoclonal anti-GFAP	1:400	1:1000	Sigma-Aldrich, Inc
Mouse monoclonal anti-MOG	n/a	1:5000	EMD Millipore, Co
Rat monoclonal anti-MBP	n/a	1:500	EMD Millipore, Co
Mouse monoclonal anti-CNPase	n/a	1:1000	EMD Millipore, Co
Mouse monoclonal anti-CC1	1:400	n/a	EMD Millipore, Co
Rabbit polyclonal anti-Olig2	1:200	n/a	EMD Millipore, Co
Goat polyclonal anti-Olig2	1:50	n/a	R&D Systems, Inc
Mouse monoclonal anti-viral nucleocapsid (N)	1:50	n/a	Kind gift from Dr. Julian Leibowitz (Texas A&M University College of Medicine, USA)
Mouse monoclonal anti-GAPDH	n/a	1:5000	Sigma-Aldrich, Inc

*IF* immunofluorescence, *WB* western blotting, *n/a* not used in that specific application

### Mouse Hepatitis Virus Infection of Mice

A neurotropic demyelinating strain of mouse hepatitis virus, MHV-A59, was used for infecting mice as described previously [33]. Briefly, 4-week-old C57BL/6 male mice were inoculated intracranially with 2000 plaque-forming units (pfu) of MHV-A59 (50% LD50 dose). Mock-infected controls were inoculated similarly with the vehicle containing phosphate-buffered saline (PBS) containing 0.75% bovine serum albumin (BSA) and maintained in parallel. Following infection, mice were monitored daily for the appearance of ruffled fur, slower movement, hindlimb paralysis, weight loss, and mortality. Animals were euthanized at 5, 15, and 30 days post-infection (pi), and spinal cord tissues were harvested as described below.

### Immunoblotting

Spinal cord tissues dissected into cervical, thoracic, and lumbosacral regions from MHV-A59 and mock-infected mice were homogenized in ice-cold RIPA lysis buffer containing EDTA-free complete protease and phosphatase inhibitors. Subsequently, tissue homogenates were centrifuged at 13,200 rpm for 20 min at 4 °C using an Eppendorf 5415R centrifuge, and the supernatants were collected. Protein concentrations were estimated using a Pierce BCA assay kit following the manufacturer’s instructions. Equal amounts of protein samples were separated on 12% SDS-PAGE or 4–12% NuPAGE Bis–Tris gels and transferred onto PVDF membranes, blocked with 5% non-fat milk in Tris-buffered saline containing 0.1% Tween-20 for 1 h at room temperature (RT), and then incubated overnight at 4 °C with either anti-Cx47, anti-Cx43, anti-CNPase, anti-MBP, anti-MOG, or anti-GFAP antisera at dilutions listed in Table 1. On the following day, membranes were washed and incubated with appropriate HRP-conjugated secondary antibodies (1:10,000), and immunoreactive proteins were visualized using SuperSignal West Pico PLUS Chemiluminescent detection kit in ChemiDoc XRS + gel imaging system (Bio-Rad Laboratories, Inc.). All blots were re-probed with anti-GAPDH antibody to monitor equal protein loading. Densitometric analysis of the blots was performed using ImageJ (NIH) software [34].

### Immunofluorescence Staining and Confocal Microscopy on Mouse Spinal Cord Cryosections

MHV-A59 and mock-infected mice at 5 and 30 days pi were transcardially perfused with 1X phosphate-buffered saline (PBS) followed with 4% paraformaldehyde (PFA) in 1X PBS. Spinal cord tissues were further post-fixed in 4% PFA for 16–18 h at 4 °C and then cryo-protected by incubating in 30% sucrose in PBS solution at 4 °C. Tissues were embedded in OCT medium and sectioned coronally on a cryostat (Leica Biosystems) to 10 µm thickness and collected on charged glass slides. Immunostaining was done as described previously [33]. Briefly, the cryosections were thawed at room temperature (RT) for 10 min and treated with chilled methanol for 10 min at − 20 °C. The sections were washed with 1X PBS solution and incubated with 0.1 M PBS-glycine for 1 h at RT. After permeabilizing the tissue sections with 0.4% Triton X-100 for 20 min at RT, blocking was done with 3% BSA and 0.25% Triton X-100 in 1X PBS for 1 h at RT. Tissue sections were incubated with primary antibody in antibody diluent containing 0.25% BSA and 0.1% Triton X-100 in 1X PBS for overnight at 4 °C in a humidified chamber. The following day, the sections were washed and incubated with appropriate secondary antibodies conjugated to Alexa Fluor 488, Alexa Fluor 568, or Alexa Fluor 647 (1:500) for 2 h at RT. All incubations were carried out in a humidified chamber. After washing, sections were mounted with Prolong Diamond antifade mounting media containing DAPI. Alexa Fluor 488 Tyramide Super Boost Kit was used for the detection of anti-Olig2 antibody staining following the manufacturer’s instructions. Confocal imaging was performed either using a Leica SP8 confocal laser scanning microscope (Leica Microsystems) equipped with a HC PL APO × 63 oil immersion objective lens or a Nikon Ti2 Eclipse confocal microscope (Nikon Corp.) equipped with a Nikon N PLAN APO 60X/1.42 oil immersion objective lens. In both instruments, imaging was performed using 405-, 488-, 552-, and 647-nm laser lines. Images were acquired and processed using LasX software (Leica Microsystems) or NIS-Elements software (Nikon Corp.), respectively. Image analysis was done using ImageJ software [34].

### Quantification of Demyelinating Lesions

To examine the loss of myelin upon viral infection, cervical and thoracic spinal cord cryosections from MHV-A59 and mock-infected mice collected at 30 days pi were labeled with a FluoroMyelin red fluorescent myelin stain following manufacturer’s protocol and imaged using EVOS-M5000 fluorescence microscope. To determine the total white matter area and areas with myelin loss, for each spinal cord region, 4–5 FluoroMyelin-stained spinal cord cross-sections from each animal were analyzed using ImageJ software. The total perimeter of the white matter regions in each section was outlined and calculated by summing the dorsal, ventral, and lateral white matter areas in each section. The total area of the demyelinated regions was also outlined and summed for each section separately. The percentage area of spinal cord demyelination per section was obtained by dividing the total area of the demyelinating plaque over the total area of the calculated white matter in the respective section and then multiplying by 100.

### Cx47-Cx43 Colocalization in CC1-Positive (CC1 +) Cells

Spinal cord cryosections from MHV-A59 and mock-infected mice collected at 5 and 30 days pi were triple labeled for anti-Cx43, anti-Cx47, and anti-CC1 antibodies as described above. Determination of colocalization of Cx43 and Cx47 puncta in CC1 + cells was carried out in confocal image stacks by using the ImageJ Colocalization Threshold plug-in. A region of interest (ROI) was drawn for each CC1 + cell using the freehand selection tool, and Pearson’s coefficient for Cx43 and Cx47 colocalization was calculated using the Coloc2 plug-in in ImageJ software.

### Quantification of Oligodendrocyte Cell Populations

Spinal cord cryosections from MHV-A59 and mock-infected mice at 30 days pi were double immunolabeled for anti-Olig2 and anti-CC1 antibodies as mentioned above. Fluorescence imaging was performed with an EVOS-M5000 fluorescence microscope, and the numbers of Olig2 + /CC1-, Olig2 + /CC1 + , and Olig2-/CC1 + cells were quantified by ImageJ software using a cell counter plug-in. Two to three sections each at least 30 µm apart from each spinal cord region were analyzed for each animal (*n* = 6 animals per group), and the three oligodendrocyte lineage cell populations (Olig2 + /CC1-, Olig2 + /CC1 + , and Olig2-/CC1 +) were quantified in areas exhibiting demyelination (DM) as well as in the surrounding normal appearing white matter (NAWM) areas.

### Statistical Analysis

Analyses were performed using GraphPad Prism version 9.5.1 software (GraphPad Software, Inc.). Statistical significance was determined by unpaired two-tailed Student’s *t* test for two group comparisons with a significance threshold set at *p* < 0.05. Data are presented as scatterplot graphs reporting the mean ± standard error of the mean (SEM) or standard deviation (SD) as indicated.

## Results

### Neurotropic Viral Infection Causes Profound and Region-Specific Differences in Demyelination of Mouse Spinal Cord

**Fig. 1:**
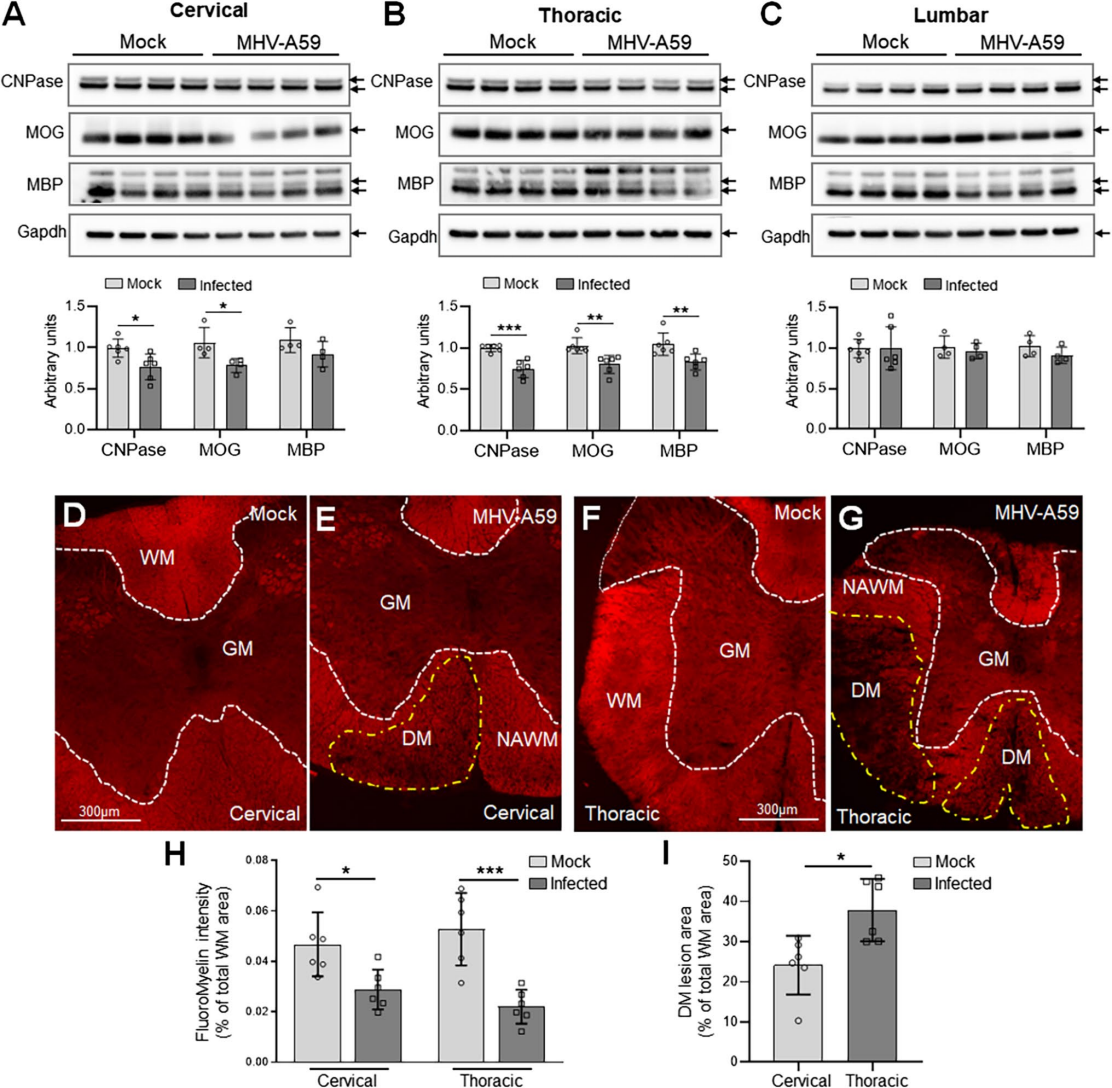
Regional variation in demyelination within the spinal cord following MHV-A59 infection at 30 days post-infection (pi). **A**–**C** Representative immunoblots and histograms depicting steady-state levels of myelin proteins CNPase (2′,3′-cyclic nucleotide 3′-phosphodiesterase, 46/48 kDa), MOG (myelin oligodendrocyte glycoprotein, 28 kDa), and MBP (myelin basic protein, 18.5–21.5 kDa) in the anterior cervical (**A**), middle thoracic (**B**), and posterior lumbosacral (C) cord regions of mock and MHV-A59-infected mice at 30 days pi. Note that only the thoracic cord of MHV-A59-infected mice shows a significant reduction of all three myelin protein levels compared to the respective control group. The cervical cord region shows significant downregulation of CNPase and MOG but not MBP, while the lumbosacral cord region does not show significant alteration of any of the myelin proteins examined. GAPDH is used as the loading control, and all values are normalized to their respective GAPDH values. Error bars denote mean ± SD of *n* = 4–6 animals/group. Each datapoint represents the mean of 2–4 technical replicates of each biological replicate. **D**–**G** Representative images showing fluorescent myelin labeling in the mock- and virus-infected cervical (**D**, **E**) and thoracic (**F**, **G**) spinal cord sections at 30 days pi. Mock-infected control tissues (**D**, **F**) show uniform myelin labeling in the white matter (WM) areas, while the MHV-A59-infected cervical (**E**) and thoracic (**G**) sections show demyelinating patches (DM) evident from the loss of fluorescent myelin labeling (areas outlined by yellow dashed lines). Note the larger area of the DM patches observed in the thoracic region compared to cervical region of the MHV-A59-infected mice (**E**, **G**). **H**, **I** Histogram depicting the FluoroMyelin intensity (**H**) in mock *versus* MHV-A59-infected spinal cord regions and the area coverage of DM lesions (**I**) in cervical *versus* thoracic spinal cord regions of MHV-A59-infected mice. Each datapoint represents the mean of the technical replicates of each biological replicate with error bars denoting mean ± SD of biological replicates (*n* = 6 animals/group). *p < 0.05, **p < 0.01, ***p < 0.001

Four-week-old C57BL/6 mice were inoculated intracranially with a neurotropic strain (A59) of mouse hepatitis virus (MHV) as described previously [33]. In keeping with earlier studies, during the acute infection stage, that is, the first week post-infection (pi), mice exhibited characteristic weight loss, hunchback, ruffled fur, reduced movement, and hindlimb paralysis. As expected by 10–14 days pi following viral clearance, the mice began regaining body weight and normal activity levels. By 30 days pi, which marks the peak of demyelinating pathology, all MHV-A59 infected mice had recovered to similar weights as mock-infected control animals and exhibited normal body posture and hindlimb movement. As reported earlier, myelin staining of the spinal cord sections showed significant loss of white matter myelination at 30 days pi (Fig. 1). Consistent with earlier findings that indicate oligodendrocytes as the primary targets of demyelination, our results also suggest that the demyelinating strain MHV-A59 can directly infect oligodendrocytes in the CNS (Fig. S1) [4, 35–37]. To further understand the demyelination profile in different spinal cord regions with increasing time pi, we examined the steady-state levels of the myelin proteins, 2′,3′-cyclic nucleotide 3′-phosphodiesterase (CNPase), myelin oligodendrocyte glycoprotein (MOG), and myelin basic protein (MBP) in cervical, thoracic, and lumbosacral regions of the spinal cord at 5, 15, and 30 days pi (Fig. 1 and Fig. S2). Immunoblot analysis indicated clear regional differences in the regulation of myelin protein levels in MHV-A59-infected mice compared to their respective mock-infected controls with the thoracic cord region showing the most consistent downregulation of the myelin proteins CNPase, MOG, and MBP at 30 days pi. Thus, while the cervical cord showed significant downregulation of CNPase and MOG only but not MBP, the thoracic region of the spinal cord showed significant downregulation of all three myelin proteins at the peak of demyelinating disease (Fig. 1A, B). Surprisingly, none of the myelin protein levels was found to be significantly altered in the lumbosacral region at this stage (Fig. 1C). The earlier time points of 5 and 15 days pi did not show any significant alteration in CNPase, MOG, and MBP protein levels in either cervical, thoracic, or lumbosacral cord regions (Fig. S2). Supporting our immunoblotting data, FluoroMyelin staining of spinal cord sections from MHV-A59-infected mice at 30 days pi showed prominent loss of myelin staining in the white matter areas of cervical and thoracic cord regions compared to the respective control sections (Fig. 1D–H). Interestingly, analysis of the area coverage of the demyelinating lesions suggested regional differences between cervical and thoracic cord sections at 30 days pi. The area affected by prominent demyelination was observed to be significantly greater in the thoracic sections compared to the cervical sections of MHV-A59-infected mice (Fig. 1I).

### Altered Dynamics of Oligodendroglial Cx47 Expression in Normal Appearing Gray and White Matter Regions and White Matter Lesions at Chronic Demyelination

**Fig. 2:**
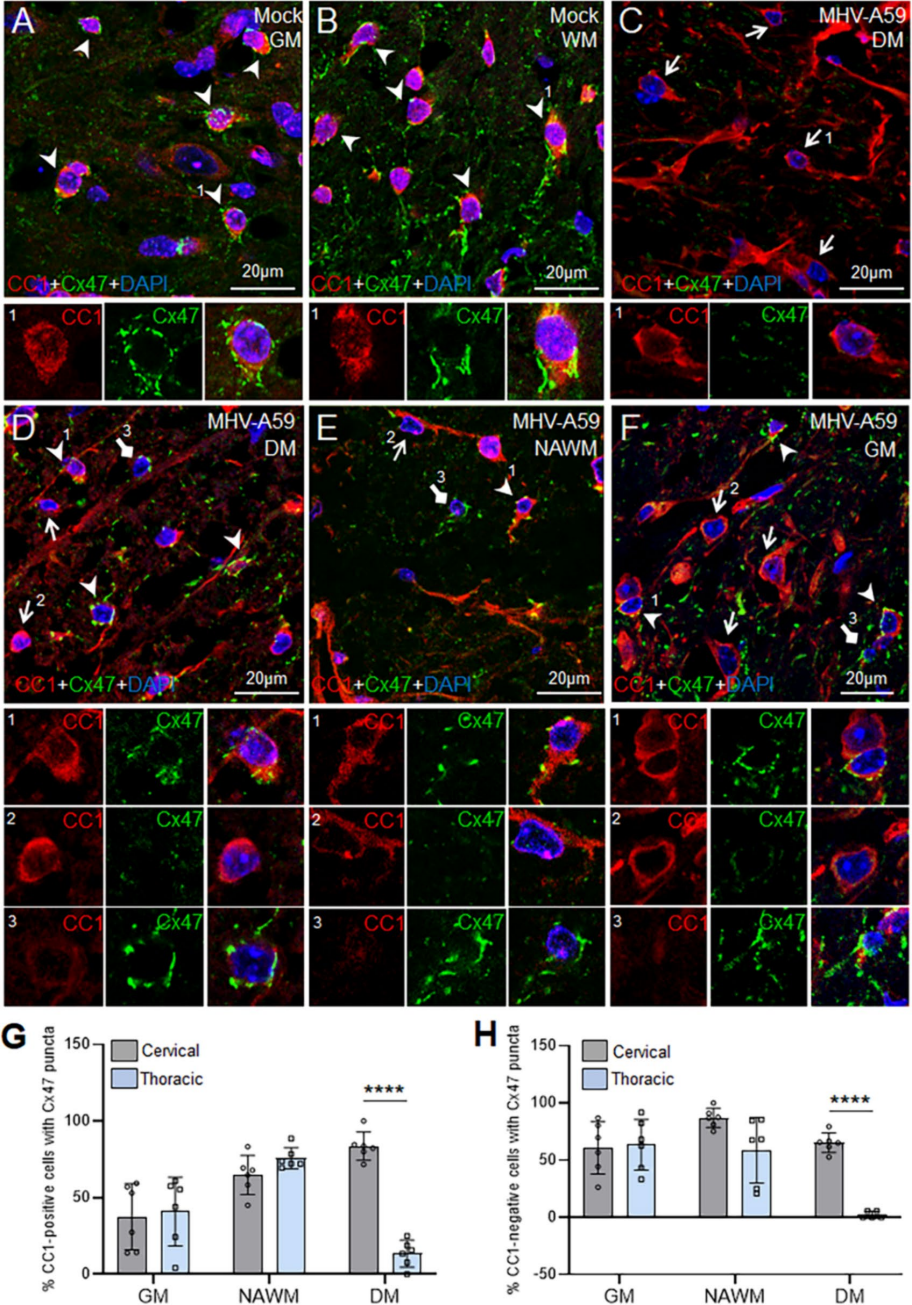
Altered Cx47 GJ expression in spinal cord oligodendrocytes (OLs) upon MHV-A59 infection at 30 days post-infection (pi). **A**–**F** Representative confocal images from gray and white matter (GM and WM, respectively) areas of spinal cord sections from mock- (**A**, **B**) and virus-infected mice (**C**–**F**) at 30 days pi immunostained for anti-Cx47 and anti-CC1 antibodies. Nuclei are stained with DAPI. Mock-infected cervical spinal cord sections (**A**, **B**) show Cx47 GJs mostly in the form of a dense ring outlining the periphery of CC1-labeled mature OLs in the GM (**A**) and WM (**B**) areas. In contrast, thoracic DM lesions exhibit a drastic reduction in Cx47 GJ staining (**C**), while the cervical DM lesions (**D**) exhibit limited Cx47 GJ expression only in some mature CC1-positive (CC1 +) OLs (arrowheads and arrow, insets 1 and 2). Note that in the cervical DM, some Cx47 GJ expression is also evident in CC1-negative (CC1-) cells (diamond arrow, inset 3). MHV-A59-infected NAWM (**E**) and GM (**F**) areas of the spinal cord show reduced Cx47 GJ expression with only some CC1 + mature OLs exhibiting Cx47 puncta (arrowheads, inset 1) while many mature CC1 + OLs having no Cx47 expression (arrows, inset 2). Further, Cx47 GJ staining is also evident in some CC1- cells (diamond arrows, inset 3) in MHV-A59-infected NAWM (**E**) and GM (**F**) areas of the thoracic cord, which is not evident in the mock-infected control sections (**A**, **B**). Arrowheads denote CC1-labeled mature OLs exhibiting Cx47 GJs, arrows denote CC1-labeled mature OLs without any Cx47 GJ puncta, and diamond arrows indicate CC1- cells with Cx47 GJs (**D**–**F**). **G**, **H** Histograms depicting the percentage of CC1 + (**G**) and CC1- (**H**) cells that stained positive for Cx47 GJs in the cervical and thoracic spinal cord of MHV-A59-infected mice at 30 days pi. Considering 100% of CC1 + OLs stained positive for Cx47 GJ expression and no Cx47 GJs were observed in CC1- cells in mock-infected controls (not plotted in the graphs), note the decrease in the percentage of CC1 + mature OLs exhibiting Cx47 GJ labeling (**G**) and increased percentage of CC1- OL cells exhibiting Cx47 GJs (**H**) in different spinal cord areas upon MHV-A59 infection at 30 days pi. Error bars denote the mean ± SD of the biological replicates (*n* = 6 animals/group). ****p < 0.0001. GM, gray matter; WM, white matter; NAWM, normal appearing white matter; DM, demyelination

To evaluate if the observed regional differences in myelin loss could be attributed to disruption of oligodendrocyte GJ formation, we investigated the expression of Cx47 in CC1-labeled mature oligodendrocytes (MOLs) in different spinal cord areas including the normal appearing gray and white matter (GM and NAWM, respectively) regions and demyelinating (DM) lesions of cervical and thoracic spinal cord sections (Fig. 2 and Fig. S3). Cx47 puncta, typically representing oligodendrocyte GJs, is normally expressed in cell bodies and proximal processes of all mature CC1-stained oligodendrocytes in mock-infected control spinal cord sections (Fig. 2A, B and Fig. S3; arrowheads). In contrast, spinal cord regions from MHV-A59-infected mice showed a marked reduction of Cx47 GJ puncta in CC1-labeled MOLs present not only in DM lesions but also in the surrounding normal appearing WM and GM areas (Fig. 2C–G). Interestingly, the percentage of CC1-labeled MOLs expressing Cx47 GJ puncta (CC1 + /Cx47 +) was found to be significantly reduced in DM areas of the thoracic spinal cord compared to the DM regions of the cervical cord region of MHV-A59-infected mice (Fig. 2C, D, and G).

On the contrary, we observed an increased expression of Cx47 GJ puncta in cells not labeled by CC1 antibody (CC1-/Cx47 +) in spinal cord sections of MHV-A59-infected mice (Fig. 2D–F, diamond arrows and inset 3). These CC1-/Cx47 + cells were not obvious in either GM or WM areas of the spinal cord sections from control animals (Fig. 2A, B and Fig. S3). The CC1-negative cells expressing Cx47 GJ puncta possibly represented the oligodendrocyte precursor cells in MHV-A59-infected spinal cord regions. We thus confirmed their oligodendroglial lineage identity by staining with Olig2, a pan-oligodendroglial marker (Fig. S4). We further quantified the percentage of CC1-negative cells expressing Cx47 GJ puncta in mock- and virus-infected spinal cord areas (Fig. 2H). While no Cx47 GJ puncta was observed in CC1-negative cells in the mock-infected controls (not plotted in the graph), all spinal cord areas from infected MHV-A59 mice, except the thoracic DM region, showed at least 50% of the CC1-negative cells expressing Cx47 GJ puncta. The DM lesions of thoracic cord regions showed very few CC1-negative cells expressing Cx47 GJs compared to those of cervical cord regions from MHV-A59-infected mice (Fig. 2C, D, and H).

**Fig. 3:**
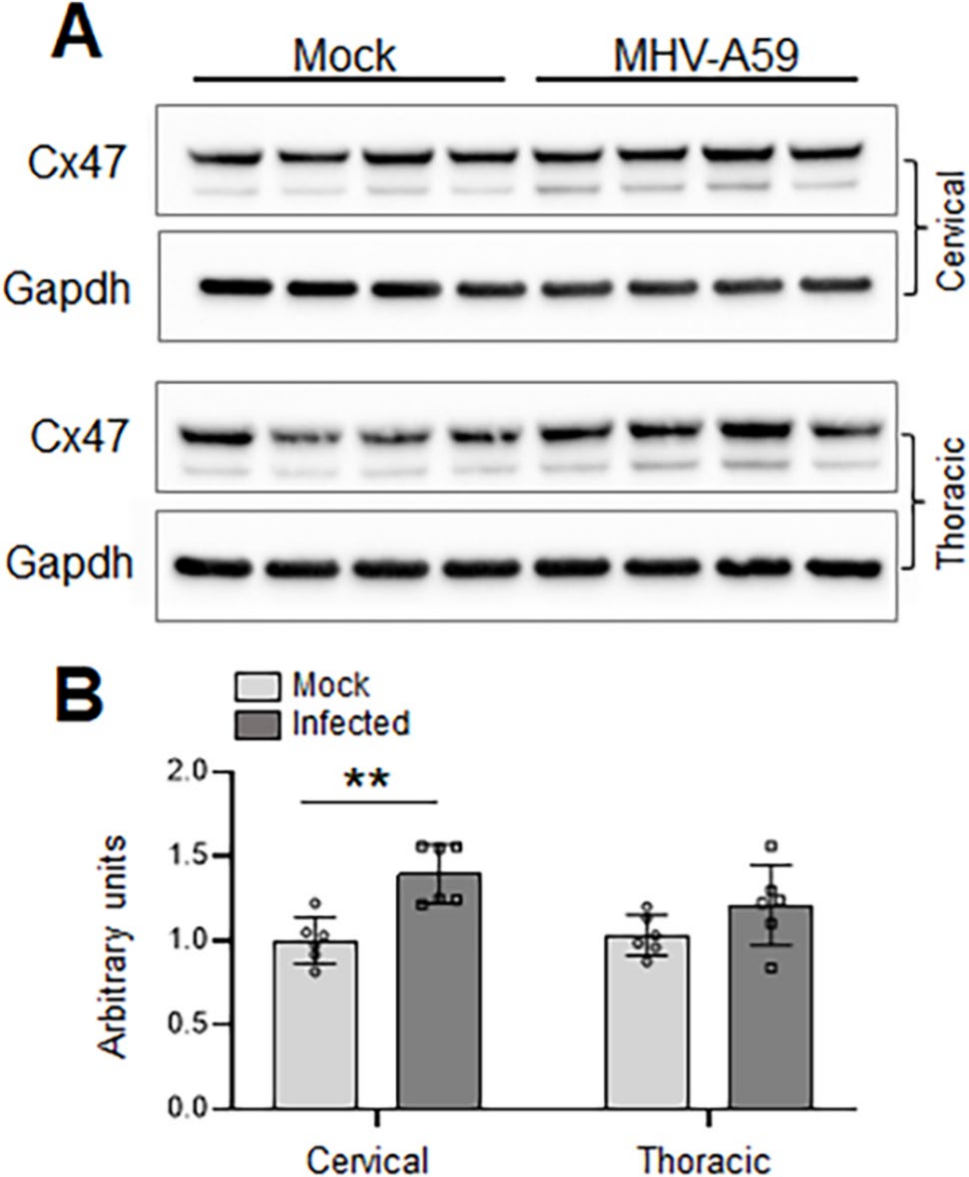
Connexin 47 (Cx47) steady-state protein levels in cervical versus thoracic spinal cord tissue following MHV-A59 infection. **A**, **B** Representative immunoblots and histograms depicting steady-state levels of Cx47 at 30 days post-infection (pi) in cervical and thoracic spinal cords of MHV-A59-infected mice compared to their respective age-matched mock-infected controls. Note that following MHV-A59 infection, the cervical cord region shows a significant increase in Cx47 protein level, while the thoracic cord shows an increasing trend in Cx47 levels compared to their respective mock-infected controls. GAPDH is used as the loading control. Error bars denote mean ± SEM of *n* = 4–6 animals/group. **p < 0.01

We further investigated if the loss of Cx47 GJ puncta is due to reduced Cx47 steady-state protein levels in cervical and thoracic spinal cord tissues during the chronic demyelinating stage (Fig. 3). Surprisingly, Cx47 protein levels were observed to be significantly increased in the cervical spinal cord tissues, while the thoracic spinal cord region showed an increasing trend without reaching statistical significance compared to their respective mock-infected controls at 30 days pi. Thus, our quantitative immunoblot analysis suggested that the loss of Cx47 GJ expression may not be due to reduced steady-state Cx47 protein levels in the spinal cord following MHV-A59 infection.

### Loss of Astroglial Cx43 and Disruption of Heterotypic O/A GJ Connectivity During Acute Infection

**Fig. 4:**
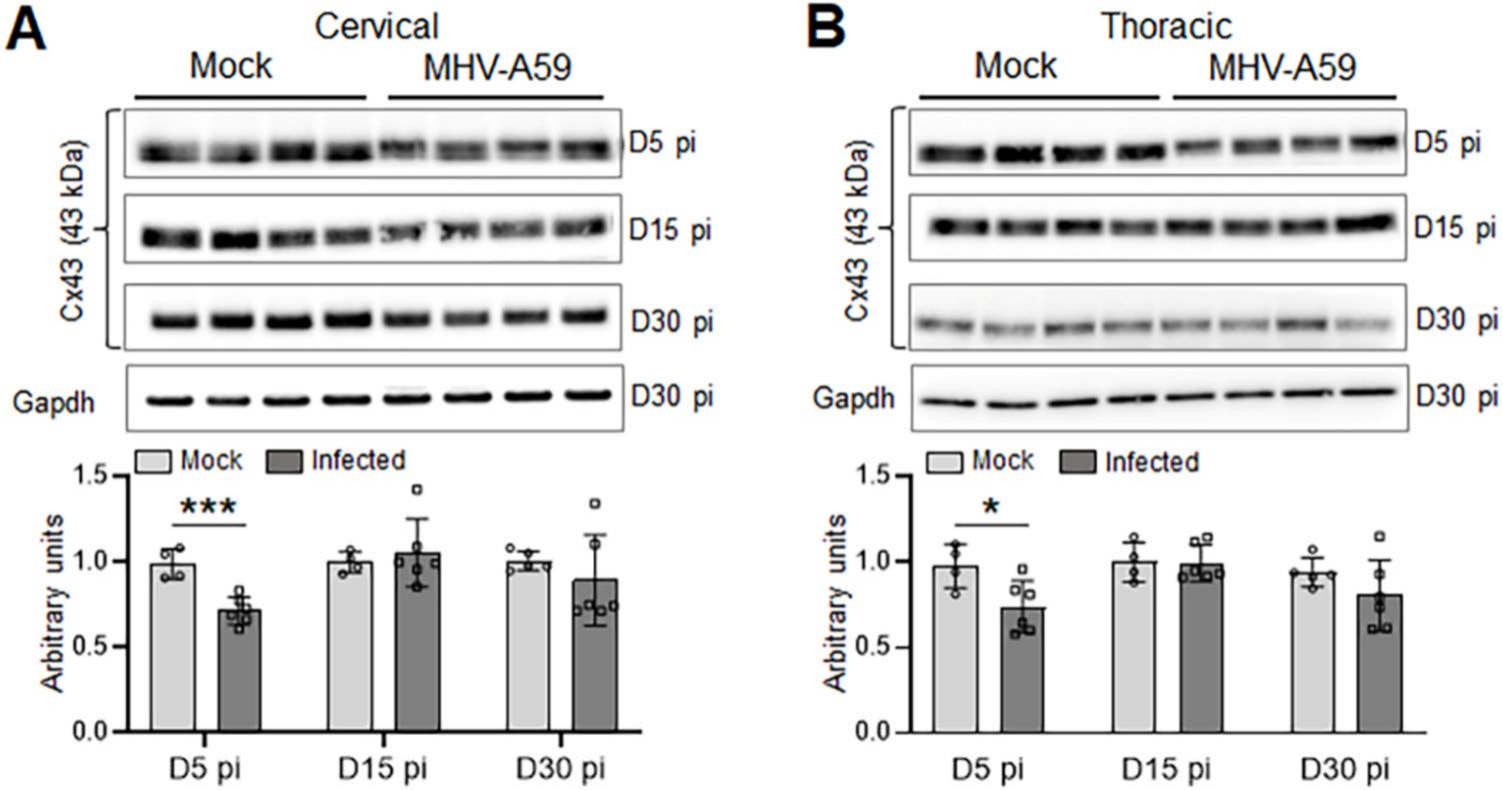
Alterations in Cx43 protein levels in cervical and thoracic spinal cord following MHV-A59 infection. **A**, **B** Representative immunoblots and histograms depicting steady-state levels of Cx43 at 5, 15, and 30 days post-infection (pi) in cervical (**A**) and thoracic (**B**) spinal cords of MHV-A59-infected mice compared to their respective age-matched mock-infected control groups. Note the significant downregulation of Cx43 protein levels during acute infection (5 days pi) which is restored back to similar levels as controls by 15 days pi. GAPDH is used as the loading control. Error bars denote mean ± SEM of *n* = 4–6 animals/group. *p < 0.05, ***p < 0.001

**Fig. 5:**
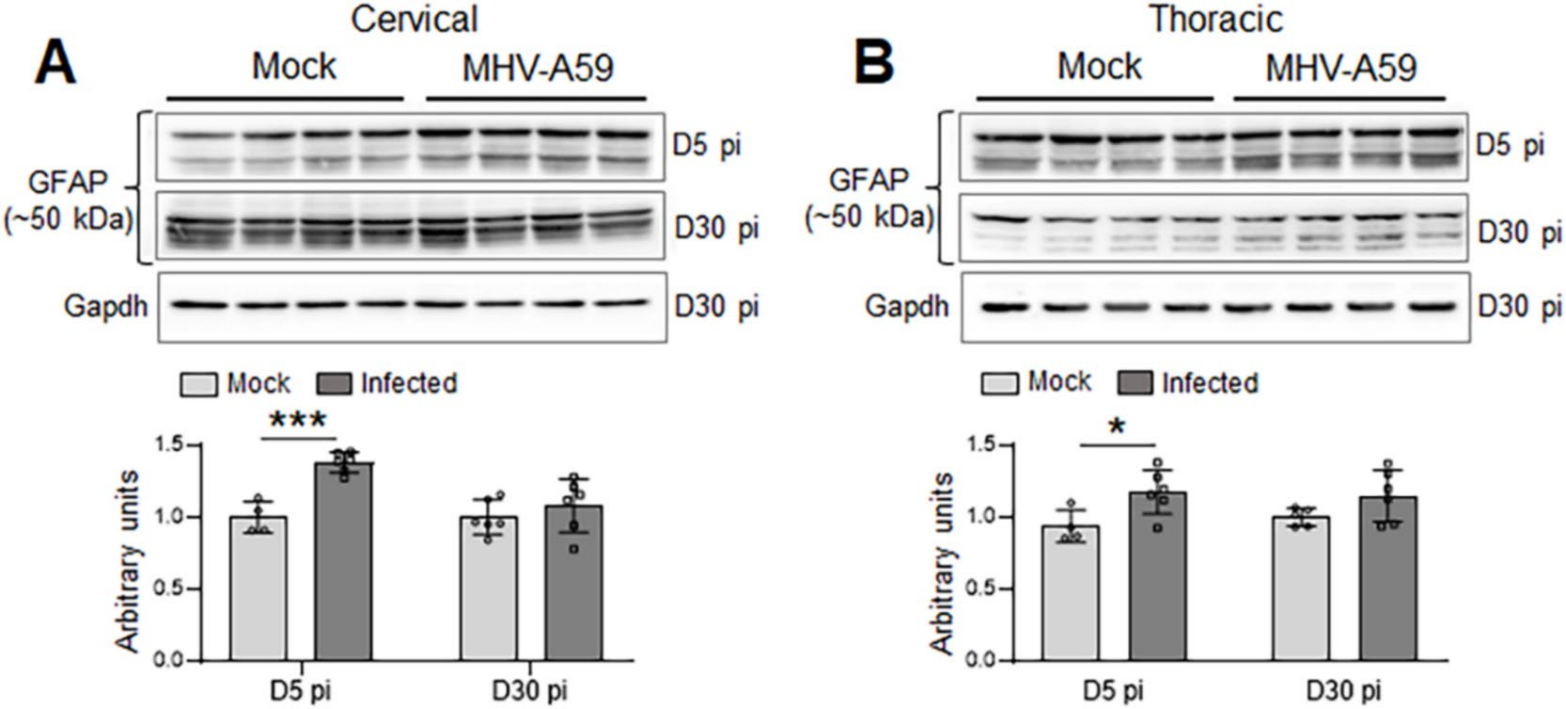
Alterations in GFAP protein levels in cervical and thoracic spinal cord following MHV-A59 infection. **A**, **B** Representative immunoblots and histograms depicting steady-state levels of GFAP at 5 and 30 days post-infection (pi) in cervical (**A**) and thoracic (**B**) spinal cords of MHV-A59-infected mice compared to their respective age-matched mock-infected control groups. GAPDH is used as the loading control. GFAP levels show a significant upregulation in both cervical and thoracic cord tissues during the acute infection at 5 days pi indicating reactive gliosis. Error bars denote mean ± SEM of *n* = 4–6 animals/group. *p < 0.05, ***p < 0.001

**Fig. 6:**
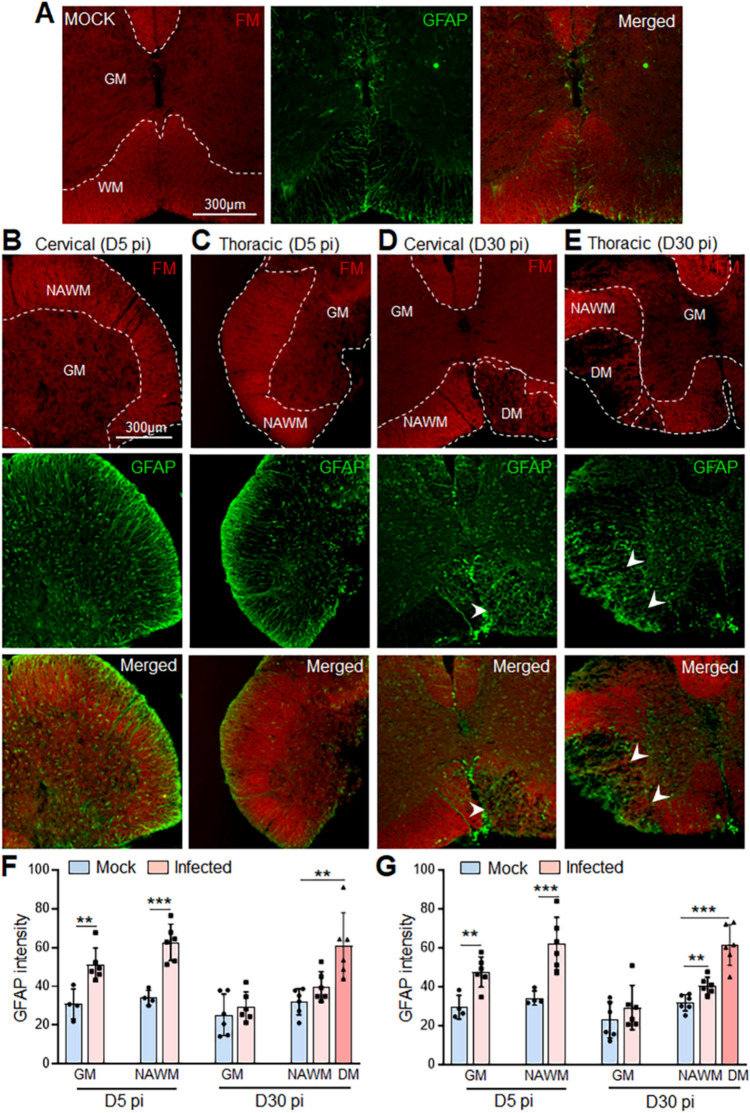
Reactive astrogliosis in the cervical and thoracic spinal cord following MHV-A59 infection. **A**–**E** Representative fluorescent images of cervical and thoracic spinal cord sections from mock- (**A**) and virus-infected mice (**B**–**E**) at 5 and 30 days post-infection (pi) immunostained for anti-GFAP antibody and counterstained with FluoroMyelin (FM). MHV-A59-infected spinal cord shows upregulated GFAP expression with immunoreactive process extending throughout the white matter (WM) areas and enlarged cell bodies in the gray matter (GM) areas of the cervical and thoracic spinal cord (**B**, **C**) during acute infection at 5 days pi compared to mock-infected control spinal cord (**A**). During chronic demyelination at 30 days pi (**D**, **E**), GFAP expression is much reduced with GFAP immunoreactive processes covering the demyelinating lesions only (arrowheads). **F**, **G** Histograms depicting the quantification of GFAP intensity in cervical (**F**) and thoracic (**G**) spinal cord sections from mock- and virus-infected mice at 5 and 30 days pi. Error bars denote the mean ± SD of the biological replicates (*n* = 6 animals/group). *p < 0.05, **p < 0.01, ***p < 0.001. GM, gray matter; WM, white matter; NAWM, normal appearing white matter; DM, demyelination

To further clarify the cause of the extensive loss of Cx47 GJs observed in CC1 + oligodendrocytes during chronic demyelination, we investigated the expression of Cx43, the main astrocytic partner of Cx47. Quantitative immunoblot analysis of Cx43 protein levels in cervical and thoracic spinal cord tissues at 5 days pi clearly indicated a significant reduction of Cx43 in MHV-A59-infected mice compared to mock-infected controls, suggesting that downregulation of Cx43 precedes the loss of Cx47 GJs from CC1 + oligodendrocytes (Fig. 4A, B). However, Cx43 protein levels were restored back to normal levels similar to mock-infected controls by 15 days pi and maintained during chronic demyelination (30 days pi). This disruption of Cx43 expression was not associated with the loss of astrocytes as GFAP levels were found to be significantly upregulated at 5 days pi and restored back to levels almost similar to controls by 30 days pi (Fig. 5A, B and Fig. S5). This was further evident in our immunostaining studies showing profound reactive astrogliosis in both cervical and thoracic cord sections at 5 days pi and to a lesser extent at 30 days pi where GFAP immunoreactive astrocytic processes were mostly restricted to the DM lesions only (Fig. 6A–G).

**Fig. 7:**
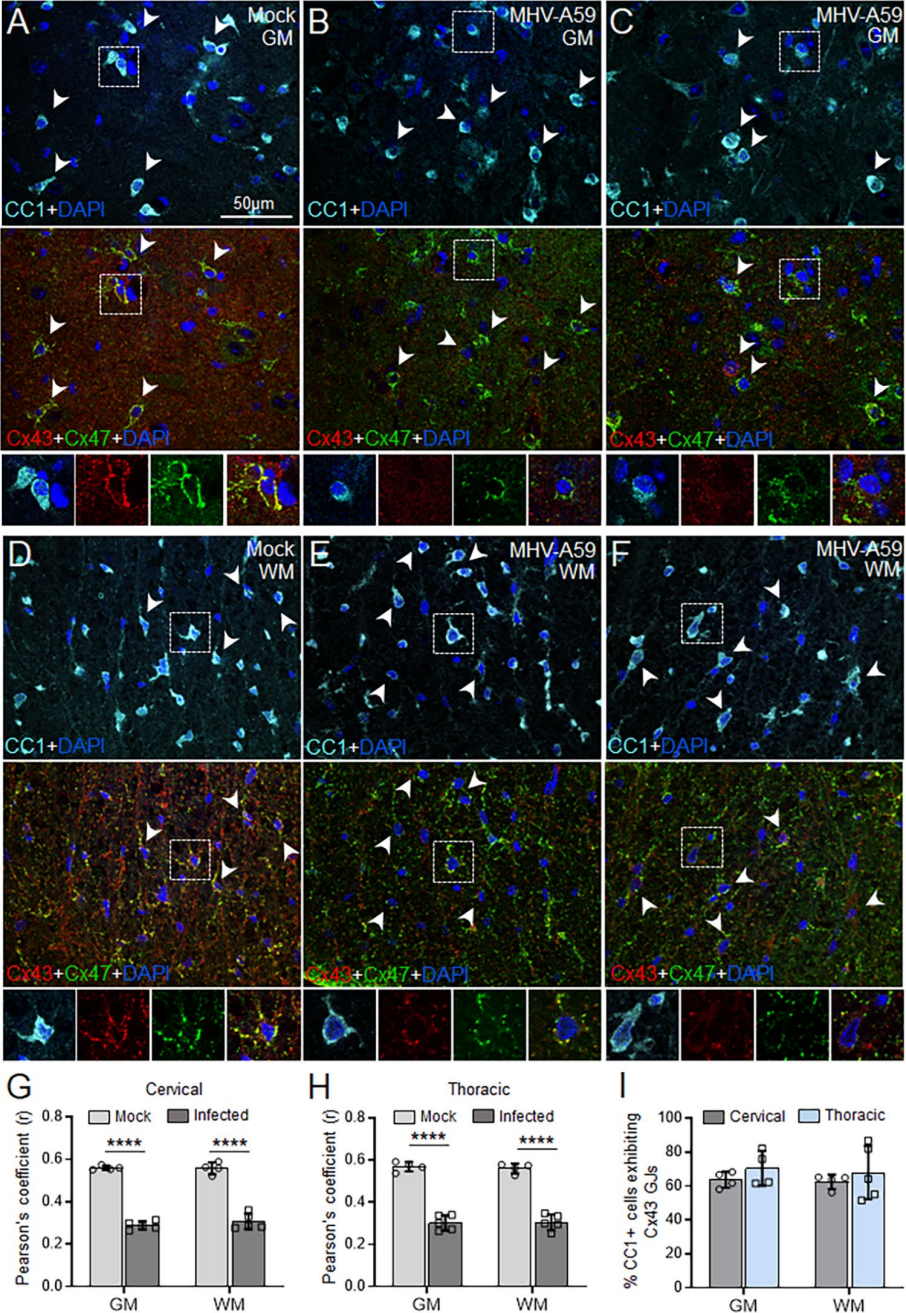
Loss of astroglial Cx43 leading to reduced Cx43-Cx47 astrocyte-oligodendrocyte GJs in mature oligodendrocytes (OLs) during acute MHV-A59 infection. **A**–**F** Representative confocal images of gray (GM, **A**–**C**) and white matter (WM, **D**–**F**) areas of mock- (**A**, **D**) and virus-infected spinal cord sections (**B**, **C**, **E**, and **F**) at 5 days post-infection triple immunostained for anti-Cx47, anti-Cx43, and anti-CC1 antibodies. Cx43-Cx47 GJs typically outline the periphery of CC1-labeled mature OLs (arrowheads) in the control cervical spinal cord (**A**, **D**). Cervical (**B**, **E**) and thoracic (**C**, **F**) cord regions show reduced Cx43-Cx47 GJ coupling due to loss of Cx43 expression (insets). **G**, **H** Plots depicting quantification of Cx43-Cx47 colocalization measured by Pearson’s correlation coefficient (*r*) using ImageJ software in gray and white matter areas of cervical (**G**) and thoracic (**H**) spinal cord. **I** Histogram showing a reduced number of CC1-labeled mature OLs exhibits Cx43 puncta during the acute infection considering all CC1-labeled cells exhibit Cx43 GJs in the control group. Each datapoint represents the mean of the technical replicates of each biological replicate (*n* = 4–5 mice/group). Error bars denote the mean ± SD of the biological replicates. ****p < 0.0001

Consequently, triple immunostaining for Cx43, Cx47, and CC1 revealed a marked loss of Cx43-Cx47 colocalization in CC1 + oligodendrocytes in both gray and white matter areas of the spinal cord in MHV-A59-infected mice compared to controls at the acute infection stage (5 days pi, Fig. 7A–H). Cx43 GJ puncta that usually appeared as dense rings outlining the CC1 + oligodendrocyte cell bodies and colocalized with Cx47 in control sections (Fig. 7A, D and Fig. S6) were markedly reduced in the gray (Fig. 7B, C) and white matter (Fig. 7E, F) areas of the infected cervical and thoracic spinal cords at 5 days pi (Fig. 7A–H). In some instances, Cx43 immunoreactivity was entirely absent in the mature OLs with only about 60% of the CC1 + cells showing the presence of Cx43 puncta in the infected spinal cord regions, in contrast to the control regions where all CC1 + oligodendrocytes exhibited Cx43 GJs (Fig. 7I). Interestingly, Cx47 GJ plaques that normally appear denser around the periphery of CC1 + oligodendrocyte cell bodies in the control tissue (Fig. 7A, D) showed a patchy appearance in many instances in MHV-A59-infected spinal cord sections, possibly indicating destabilization of Cx47 GJs following the loss of Cx43 from O/A junctions during acute infection at 5 days pi (Fig. 7C, inset).

### Persistent Loss of Heterotypic Cx47-Cx43 O/A GJ Expression at Chronic Demyelination

**Fig. 8:**
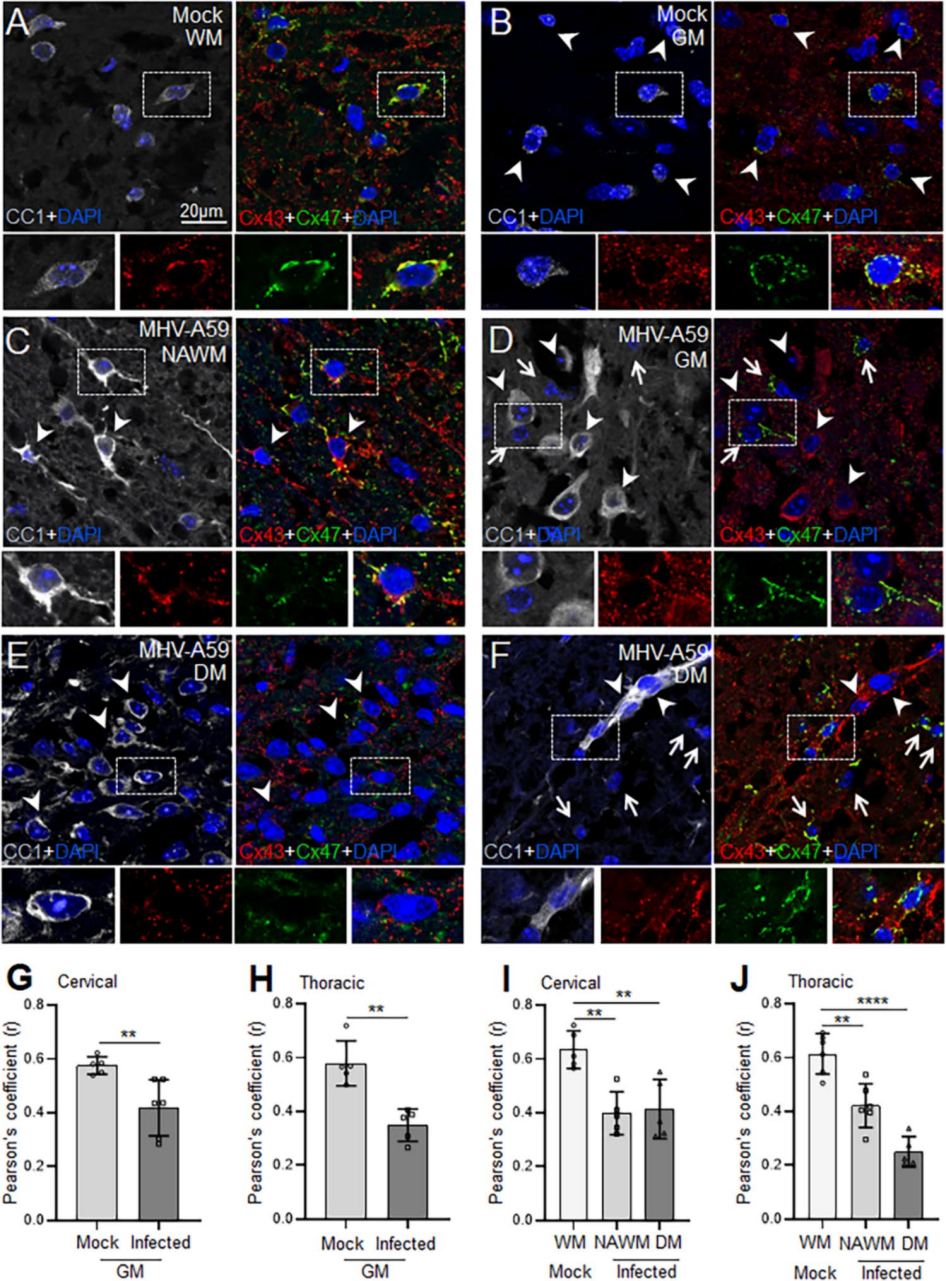
Altered Cx43-Cx47 astrocyte-oligodendrocyte GJ expression upon MHV-A59 infection at chronic demyelination. **A**–**F** Representative confocal images from mock- (**A**, **B**) and virus-infected spinal cord sections (**C**–**F**) at 30 days post-infection (pi) triple immunostained for anti-Cx47, anti-Cx43, and anti-CC1 antibodies. Nuclei are stained with DAPI. Note that loss of Cx43-Cx47 colocalization in CC1-labeled oligodendrocytes (arrowheads) in MHV-A59-infected thoracic NAWM, GM, and DM areas (**C**–**E**) compared to respective mock-infected control sections (**A**, **B**). Also, note that thoracic DM (**E**) shows a more prominent loss of Cx43/Cx47 labeling compared to the cervical DM area (**F**). Arrows indicate the appearance of Cx43-Cx47 GJ puncta in CC1-negative cells in infected tissue (**D**, **F**). **G**–**J** Plots depicting quantification of Cx43-Cx47 colocalization measured by Pearson’s correlation coefficient (*r*) using ImageJ software in gray (**G**, **H**) and white (**I**, **J**) matter areas of cervical and thoracic spinal cord. GM, gray matter; WM, white matter; NAWM, normal appearing white matter; DM, demyelination. Each datapoint represents the mean of the technical replicates of each biological replicate (*n* = 5–6 mice/group). Error bars denote mean ± SD. ****p < 0.0001

Given that Cx43 levels were restored back to normal levels following the clearance of the infectious virus particles (Fig. 4), we examined if the newly expressed Cx43 can re-establish O/A GJ connectivity in the spinal cord at 30 days pi. Colocalization analysis of Cx43 and Cx47 puncta in CC1 + mature oligodendrocytes in triple immunostained cervical and thoracic spinal cord sections revealed a persistent significant reduction in Cx43-Cx47 GJ expression in infected tissues at 30 days pi compared to the control group (Fig. 8A–J and Fig. S7). In control tissues, most Cx47 GJ puncta colocalized with Cx43 as dense rings outlining the periphery of CC1 + oligodendrocytes in WM and GM areas of the spinal cord (Fig. 8A, B and Fig. S7A, B). In contrast, in MHV-A59-infected NAWM and GM expression of Cx43 was associated with only partial recovery of Cx43-Cx47 GJs in CC1 + oligodendrocytes at 30 days pi (Fig. 8C, D and Fig. S7C, D). Interestingly, the DM areas in the infected thoracic spinal cord showed complete absence of Cx47 GJ immunoreactivity in CC1 + oligodendrocytes despite the presence of Cx43 puncta (Fig. 8E). On the contrary, cervical DM lesions exhibited Cx43-Cx47 GJ colocalization although at a much-reduced level compared to the respective control group (Fig. 8F). Additionally, some CC1- oligodendrocyte precursor cells exhibited Cx47 GJ immunoreactivity as is evident in the cervical NAWM and DM areas of MHV-A59-infected spinal cord (Fig. 8D, F, arrows). This Cx47 GJ immunoreactivity was observed to colocalize with Cx43 puncta, possibly indicating the formation of Cx43-Cx47 GJs in oligodendrocyte precursors, albeit to a limited extent.

### Changes in the Oligodendrocyte Populations at Chronic Demyelination

**Fig. 9:**
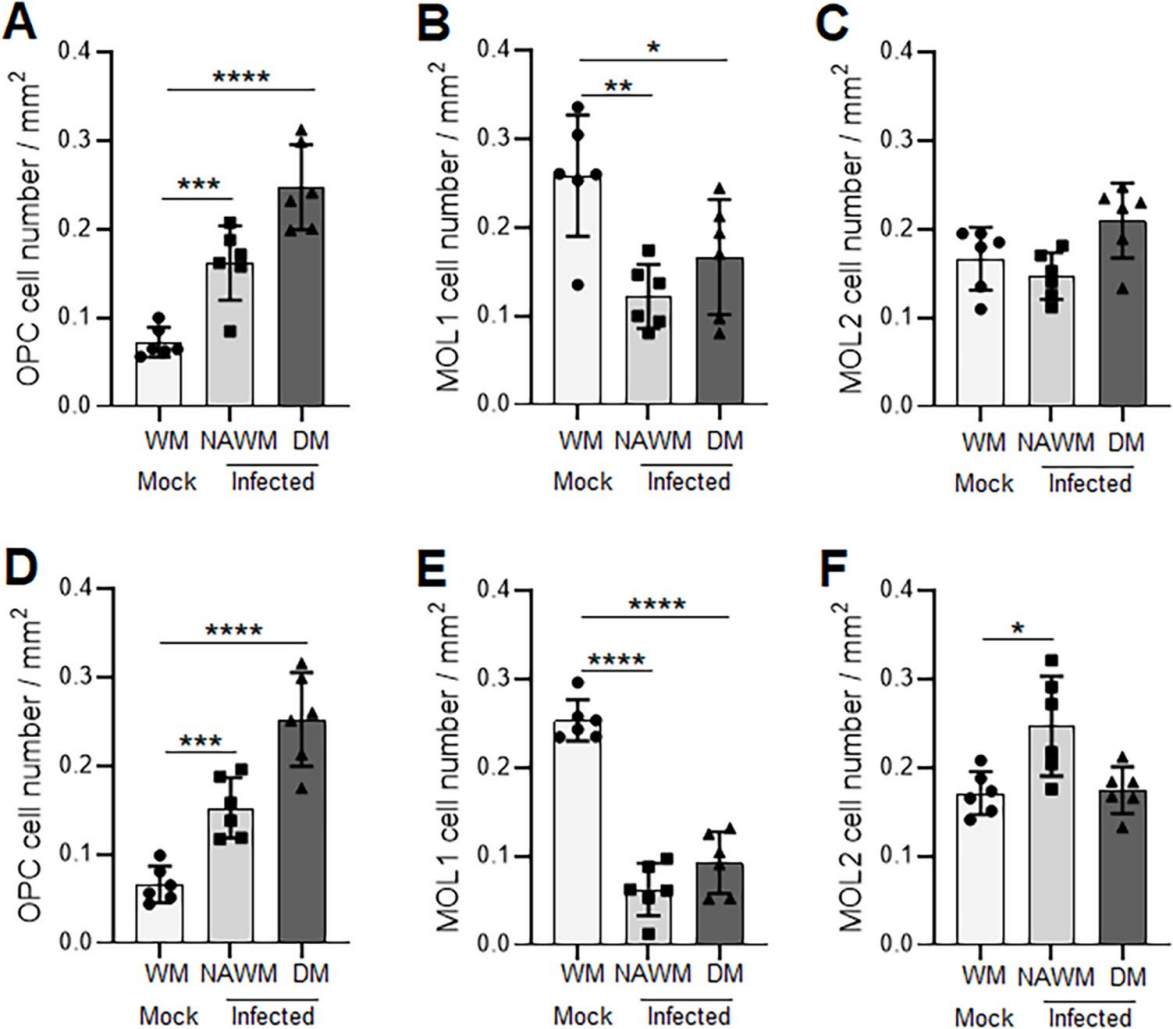
Changes in different oligodendrocyte (OL) lineage cell populations in MHV-A59-infected spinal cord at chronic demyelination. **A**–**F** Histograms depicting alterations in the number of precursor (**A**, **D**) and mature (**B**, **C**, **E**, and **F**) oligodendrocyte cell populations in the gray and white matter areas of cervical (**A**–**C**) and thoracic (**D**, **E**) spinal cord sections identified by labeling of anti-Olig2 and anti-CC1 antibodies. OPC, oligodendrocyte precursor cells denoted by Olig2 + /CC1- labeling; MOL1, mature OLs double labeled for both markers (Olig2 + /CC1 +); MOL2, mature OLs denoted by Olig2-/CC1 + labeling; GM, gray matter; WM, white matter; NAWM, normal appearing white matter; DM, demyelination. Values represent the mean of the technical replicates of each biological replicate (*n* = 6 mice/group). Error bars denote the mean ± SD of biological replicates. *p < 0.05, **p < 0.01, ***p < 0.001, and ****p < 0.0001

To further understand the observed discrepancy between Cx47 GJ expression and its steady-state protein levels and their association with the regional variation in demyelination, we investigated the proportion of different oligodendrocyte lineage cell populations at the chronic demyelinating stage. We performed doubling immunolabeling for Olig2, a pan-oligodendrocyte marker, and CC1, which specifically identifies mature OLs. We quantified three major oligodendrocyte populations in the WM areas of cervical and thoracic sections at 30 days pi, particularly the Olig2 + /CC1- oligodendrocyte precursor cells (OPCs) and Olig2 + /CC1 + and Olig2-/CC1 + mature oligodendrocytes, representing mature oligodendrocyte population 1 (MOL1) and MOL2, respectively (Fig. 9A–F and Fig. S8A–I). Our results show a significant upregulation of the OPCs in DM as well as NAWM areas of both cervical and thoracic cord regions of MHV-A59-infected spinal cords compared to the WM area of the control group (Fig. 9A, D and Fig. S8A, D, G). As expected, the MOL1 population was significantly reduced in the DM lesions of both cervical and thoracic spinal cord compared to the WM area of the respective control groups (Fig. 9B, E and Fig. S8B, E, H). Interestingly, the reduced number of MOL1 cells was not only restricted to the DM lesions but was also observed in the surrounding NAWM areas of cervical and thoracic sections (Fig. 9B, E). Moreover, the percentage decrease of MOL1 cells in the MHV-A59-infected tissue compared to its respective mock-infected control was found to be significantly greater for thoracic NAWM and DM areas than the cervical NAWM and DM regions, respectively (75.35 ± 10.07 *versus* 52.76 ± 12.76% decrease ± SD in thoracic *versus* cervical NAWM areas; 63.38 ± 13.74 *versus* 35.52 ± 25.12% decrease ± SD in thoracic *versus* cervical DM lesions). The MOL2 population of mature OLs, however, did not show any significant difference compared to the control group in the DM lesions of either cervical or thoracic cord regions, *albeit* an increasing trend in their number was noted in cervical DM lesions (Fig. 9C, F and Fig. S8C, I). Interestingly, this population of oligodendrocyte cells showed a significant increase in the NAWM of the thoracic spinal cord of MHV-A59-infected mice compared to the control group but not in the cervical NAWM (Fig. 9C, F). Thus, our data suggest significant differences in the proportion of different oligodendrocyte lineage cells that are present in cervical and thoracic cord regions at chronic demyelination that correlate with the regional variation in the extent of demyelination observed between the cervical and thoracic cord regions. Further, our data also suggest the presence of a compensatory mechanism, with an increased number of OPCs displaying Cx47 gap junctions, potentially compensating for the loss of Cx47 expression in CC1-labeled mature oligodendrocytes.

## Discussion

**Fig. 10:**
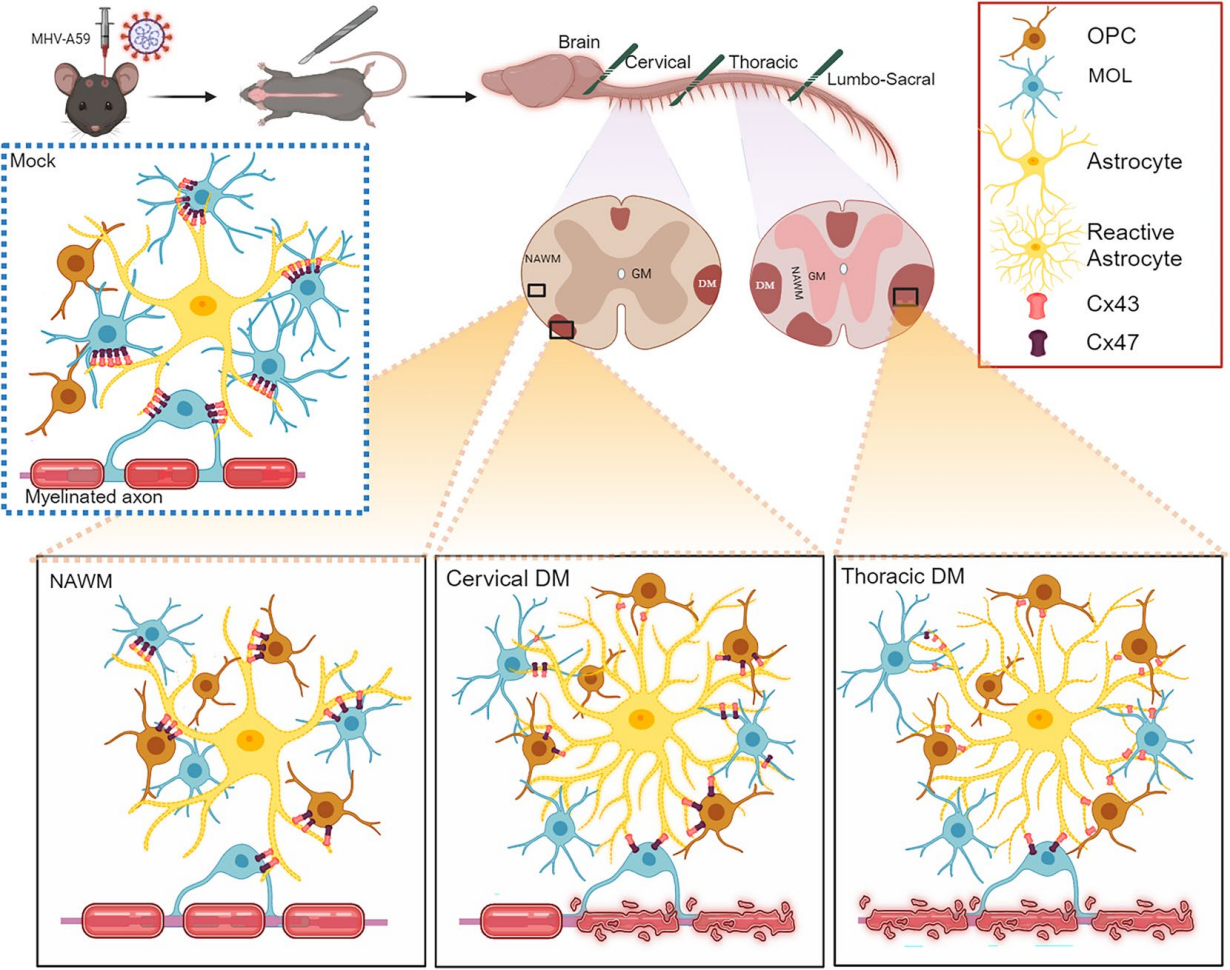
Schematic representation of the changes in Cx47-Cx43 O/A GJ expression in cervical and thoracic spinal cord regions in association with demyelination upon intracranial MHV-A59 infection. Note the dynamic shift in Cx47 GJ expression in OL lineage cell populations upon MHV infection. In mock-infected control mice, most Cx47-Cx43 GJs are observed in MOLs with no Cx47 GJ expression evident in the OPCs. Upon infection, an increase in OPCs is noted in cervical and thoracic NAWM and DM regions. Interestingly, the OPCs exhibit Cx47-Cx43 GJs in infected cervical NAWM and DM areas and in the thoracic NAWM region. On the contrary, Cx47-Cx43 GJ expression in MOL cells is significantly reduced upon infection in all areas of the cervical and thoracic spinal cord. Strikingly, the thoracic DM lesions, which are more pronounced compared to cervical DM lesions at 30 days post-infection, show complete loss of Cx47 GJ puncta in OL cells. DM, demyelination; NAWM, normal appearing white matter. Image created in BioRender.com

In this study, we have established regional differences in the extent of demyelination along the length of the spinal cord following intracerebral infection with a neurotropic strain of murine β-coronavirus, MHV-A59. The study notes the presence of varying proportions of different oligodendrocyte (OL) lineage cells including precursor and mature populations that closely associate with the demyelination profile in cervical *versus* thoracic spinal cord regions. Importantly, the data indicates differential expression of Cx47 GJs in different OL cell populations and its colocalization with its heterotypic astroglial GJ coupling partner Cx43, which may contribute to the regulation of chronic expansion of demyelinating lesions upon MHV infection (Fig. 10).

The CNS pathology induced by MHV involves a recurring pattern of demyelination and subsequent spontaneous remyelination in the spinal cords of infected mice [9, 14]. Demyelination in MHV-infected mice typically takes place during the virus clearance process, and remyelination commences around day 14 pi [4, 37–40]. The primary site of demyelination induced by MHV is the spinal cord. Areas with demyelination are distributed throughout the spinal cord, with a notable presence in the ventral portions of cervical and thoracic regions, while the lumbar region remains relatively unaffected [12, 37]. Keeping with this, our results showed significant loss of myelin protein levels most consistently in the thoracic cord region and to some extent in the cervical region but with no significant alterations in CNPase, MOG, or MBP levels in the lumbar region till 30 days pi. Additionally, our results indicate that between the cervical and thoracic spinal cord regions, the thoracic cord exhibited greater areas of demyelinating lesions at 30 days pi, as was evident from FluoroMyelin labeling, suggesting a differential regulation of demyelination and repair mechanisms among the different spinal cord regions. In most demyelinating diseases, direct targets are the OLs. Thus, these differences correlated with dynamic shifts in the proportions of precursor and mature OL lineage cell populations observed in the cervical and thoracic spinal cord at 30 days pi. As expected, Olig2 + /CC1 + (MOL1) mature OL cell populations were significantly reduced in the DM lesions of both cervical and thoracic cords compared to the respective control groups. Strikingly, this subgroup of oligodendrocytes was also significantly less in the surrounding NAWM areas of infected thoracic and cervical spinal cord tissues compared to their respective control groups with the thoracic NAWM and DM showing a more prominent decrease compared to the cervical NAWM and DM areas of infected mice.

We also observed a population of CC1 + mature OLs that were negative for Olig2 expression (Olig2-/CC1 + , MOL2). The monoclonal antibody clone CC1 is a widely used reliable marker for detecting mature oligodendrocytes in postnatal animals (older than P15 in the brain and P7 in the spinal cord) [41]. Earlier reports suggest that during early development, the Qki7 protein that binds CC1 antibody is broadly expressed in neural progenitor cells and later in their glial progeny, including OPCs and newborn astrocyte progenitor cells. However, with time, Qki expression is gradually reduced in astrocyte lineages but is strongly upregulated in differentiated oligodendrocytes. Consistent with this, we did not observe any GFAP labeling in CC1-labeled cells (Fig. S9). On the contrary, Olig2 is a crucial transcription factor vital for oligodendrocyte development, but some mature oligodendrocytes marked by CC1, particularly in response to injury or specific CNS regions, might not express Olig2 [42, 43]. Further, Olig2 deletion studies show that while continuous expression of Olig2 in mature oligodendrocytes is not necessary for myelin maintenance but is critical for remyelination after a demyelinating injury [44]. Interestingly, we detected a prominent increase in the number of these cells in the thoracic NAWM compared to WM areas of control mice. A significant decrease in Olig2 + /CC1 + (MOL1) and a corresponding increase in Olig2-/CC1 + (MOL2) mature OLs in the thoracic NAWM areas upon MHV infection might indicate dynamic changes in the differentiation and maturation states of the OLs which may determine their role in the ongoing demyelination. OLs frequently endure the acute MHV infection, persisting within the CNS even after the infection has been resolved [37]. These surviving mature OLs exhibit expression of genes associated with inflammatory response, potentially causing prolonged inflammatory alterations with CNS region-dependent impairment in remyelination [37, 45, 46]. In regions of remyelination in rodent models, both newly recruited and pre-existing mature OLs are present, but it seems that only the newly recruited ones possess the capability for remyelination [47, 48]. This study does not provide clarity on whether the two populations of CC1 + mature OLs (MOL1 and MOL2) are pre-existing or newly recruited following MHV infection and whether they can play a role in the remyelination process. However, variations in their proportions in the NAWM areas surrounding the DM lesions suggest a possible link to the ongoing demyelination.

Spontaneous remyelination is limited in demyelinating conditions like MS, primarily due to the inability of adult OPCs to differentiate into myelinating OLs. Interestingly, we detected a significant increase in the number of OPCs within DM lesions and in the surrounding NAWM of both cervical and thoracic spinal cord regions at days 30 pi. Thus, our data suggest that prior infection does not hinder OPC proliferation and migration to demyelination sites. However, the functional capacity of these newly generated OPCs, particularly their ability to differentiate into mature myelinating oligodendrocytes and contribute to remyelination, requires further exploration in subsequent studies.

This study further suggests that Cx47 GJs are lost in mature OLs during chronic demyelination. Instead, a noteworthy proportion of CC1-negative cells representing a precursor population were found to exhibit Cx47 GJ expression at the chronic demyelinating stage. Furthermore, we demonstrated that these Cx47 GJs in OPCs mostly colocalized with Cx43 in astrocytes indicating the formation of O/A GJs. Several studies have demonstrated that astrocytes, via Cx47, play a vital regulatory role in controlling OPC proliferation [49–53]. Thus, the expression of Cx47 in proliferating OPCs, following MHV-A59 infection, may play a crucial role in the generation of OPCs as part of a spontaneous effort for remyelination during the chronic demyelinating stage.

Moreover, it is well established that the stable expression of Cx47 in oligodendrocytes relies on interactions with astrocytes, as Cx47 hemichannels undergo phosphorylation and stabilization as GJ plaques by Cx43 [54]. Consequently, the loss of astroglial Cx43, as seen in acute infection (5 days pi), could lead to the disruption of O/A GJs between mature OLs and astrocytes. In fact, in many instances, we noted diffusion of Cx47 GJ puncta away from the CC1 + mature OL cell membrane giving a diffuse cytosolic appearance in the absence of astroglial Cx43 during the acute infection stage. It is possible that this diffuse cytosolic Cx47 staining in mature OLs represents internalized Cx47 GJs which may be targeted for degradation eventually leading to reduced Cx47 GJ expression in CC1-labeled mature oligodendrocytes at the chronic demyelinating stage. Notably, our findings reveal that at 30 days pi representing the chronic demyelination stage, Cx43 GJ puncta re-emerge. However, in the absence of Cx47 GJ expression in mature OLs during this stage, there is a continual loss of O/A GJ formation. In the absence of Cx47, astroglial Cx43 can exist as non-junctional hemichannels that open at the plasma membrane, releasing contents into the extracellular space. These Cx43 hemichannels can release microglia chemo-attractants and toxic molecules such as adenosine 5′-triphosphate and glutamate, along with inflammatory chemokines, contributing to adverse outcomes in the affected cellular environment [55, 56]. In fact, oligodendroglia-specific conditional Cx47 ablation in EAE mice has been shown to disrupt O/A GJs and drive CNS tissues toward a proinflammatory condition, with an enrichment of C3 + proinflammatory A1 astroglia. Such proinflammatory astroglia may contribute to stronger proinflammatory activation of microglia with increased expression of chemokines that can attract CD3 + T cells [57]. In this context, we observed pronounced upregulation of GFAP immunoreactive processes indicating ongoing reactive astrogliosis in the DM lesions characterized by loss of Cx47 GJ expression at the chronic demyelination stage. Importantly, our studies showed that the thoracic DM lesions exhibited a more pronounced downregulation of Cx47 GJ puncta compared to cervical DM lesions. Therefore, it is conceivable that chronic demyelinated lesions may be more susceptible to neuroinflammation due to the prolonged absence of Cx47 regulatory control over glial inflammation, which can contribute to unsuccessful remyelination and axon loss.

Interestingly, our study also revealed that despite the loss of Cx47 GJs in mature OLs, the Cx47 total protein levels were not reduced compared to respective mock-infected control mice. In fact, Cx47 steady-state protein levels were found to be significantly increased in cervical tissue, while the thoracic cord only showed an increasing trend without reaching statistical significance. This could be attributed to the increased Cx47 GJ expression in the CC1-negative precursor OL cell population and dynamic shifts in the proportion of precursor and mature OL cells observed in the spinal cord following intracerebral MHV-A59 infection. While we observed a decrease in Cx47 GJ expression in CC1 + mature oligodendrocytes, there was an increase in Cx47 GJ expression in CC1-negative oligodendrocyte precursor cells, in the MHV-infected cervical and thoracic spinal cords compared to respective control groups. In fact, our results align with studies conducted on human multiple sclerosis (MS) brains, where newly differentiated OLs may exhibit Cx47 GJ expression, while the presence of Cx47 in mature OLs is consistently absent in chronic MS plaques [27, 29]. Furthermore, our cell counting data showed a significant increase in number of the precursor oligodendrocytes in the cervical and thoracic spinal cord of MHV-infected mice compared to controls, which might compensate for the loss of Cx47 expression seen in mature OLs. Thus, we presume that the observed cell-state-specific dysregulation of Cx47 expression in different oligodendrocyte cells along with the presence of varying proportions of these cells in cervical and thoracic cord regions following infection might contribute to the increased steady-state Cx47 protein levels in tissue extracts.

In conclusion, this study demonstrates variance in the proportion of different OL lineage cells marked by differential Cx47 GJ expression and their connection with astroglial Cx43 associated with demyelination following a murine β-coronavirus infection. The findings indicate altered Cx47 GJ expression in different OL cell populations might be a significant factor that plays an important role in regulating the expansion of demyelinating lesions following a neurotropic viral infection.

## Supplementary Information

Below is the link to the electronic supplementary material.

Supplementary file1 (DOCX 14.8 MB)

## Data Availability

The data supporting the findings of this study are all contained within the manuscript.
