# Supplementary material for: Neurotropic Murine β-Coronavirus Infection Causes Differential Expression of Connexin 47 in Oligodendrocyte Subpopulations Associated with Demyelination
Source: Mol Neurobiol. 2024 Sep 18;62(3):3428–45. doi: 10.1007/s12035-024-04482-0 (PMC11790745; doi:10.1007/s12035-024-04482-0)
Supplement: Supplementary file 1 — Supplementary file1 (DOCX 14.8 MB) [file 12035_2024_4482_MOESM1_ESM.docx]

**Supplementary information**

**
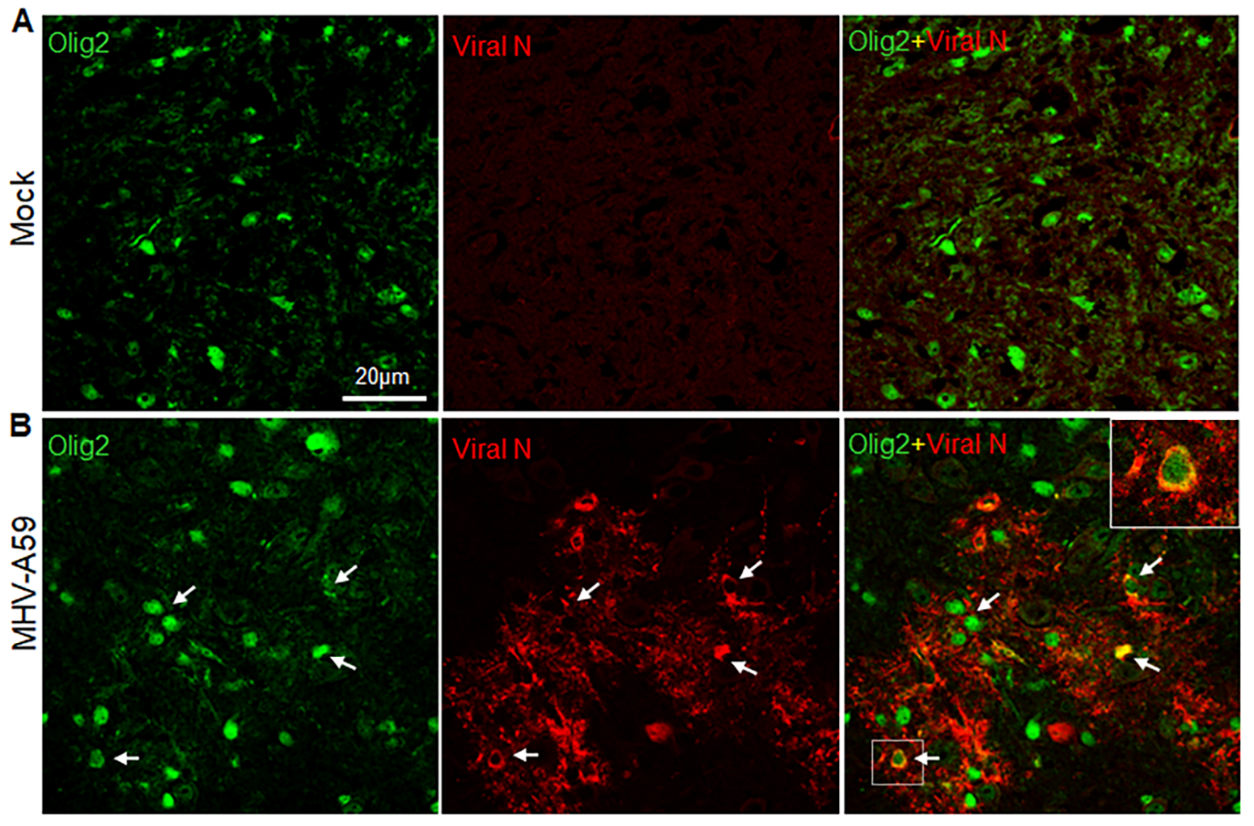
**

**Fig. S1** Representative confocal images showing direct viral infection of oligodendrocytes at 5-days post-infection. (A, B) Double immunofluorescence labelling of mock (A) and MHV-A59 (B) infected mouse spinal cord showing viral nucleocapsid (N) antigen labelling in Olig-2-positive oligodendrocytes in virus infected tissue (B). No viral N staining is evident in the control tissue sections (A).

**
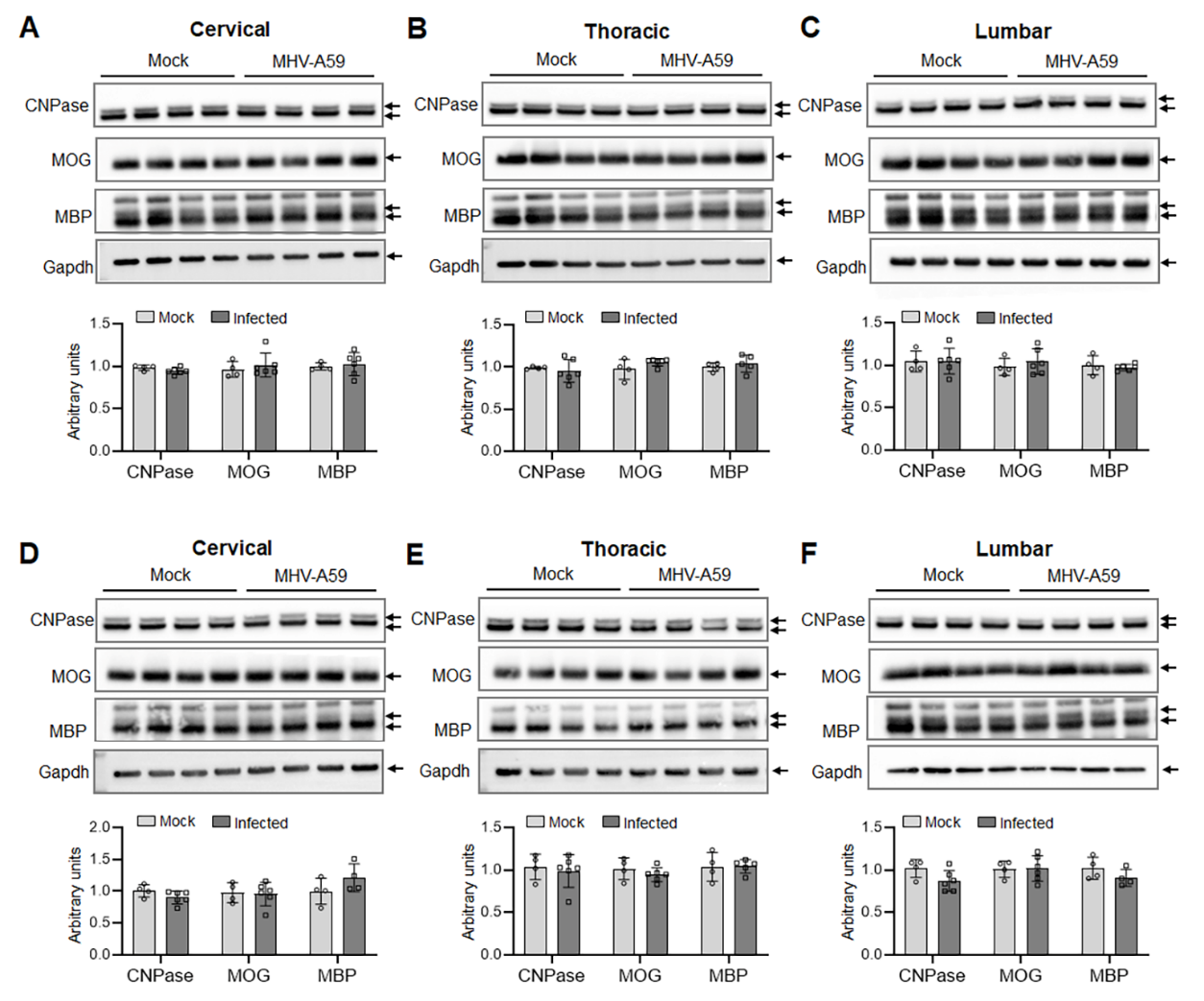
**

**Fig. S2** Steady-state myelin protein levels showing no significant alteration in MHV-A59 infected mouse spinal cord tissue at 5- and 15-days post-infection (pi) compared to respective mock-infected control regions. (A-C) Representative immunoblots and histograms depicting steady-state levels of myelin proteins CNPase (2',3'-Cyclic-nucleotide 3'-phosphodiesterase, 46/48 kDa), MOG (Myelin oligodendrocyte glycoprotein, 28 kDa) and MBP (Myelin basic protein, 18.5-21.5 kDa) in the anterior cervical (A), middle thoracic (B) and posterior lumbo-sacral (C) cord regions in mock and MHV-A59 infected mice during acute infection (5 days pi). (D-F) Representative immunoblots and histograms depicting steady-state levels of CNPase, MOG and MBP in the cervical (D), thoracic (E) and lumbo-sacral (F) regions of the spinal cord in mock and MHV-A59 infected mice at 15 days pi. Gapdh is used as the loading control and all values are normalized to their respective Gapdh values. Error bars denote mean±SD of n=4-6 animals/group. Each datapoint represents the mean of 2-4 technical replicates of each biological replicate.

**
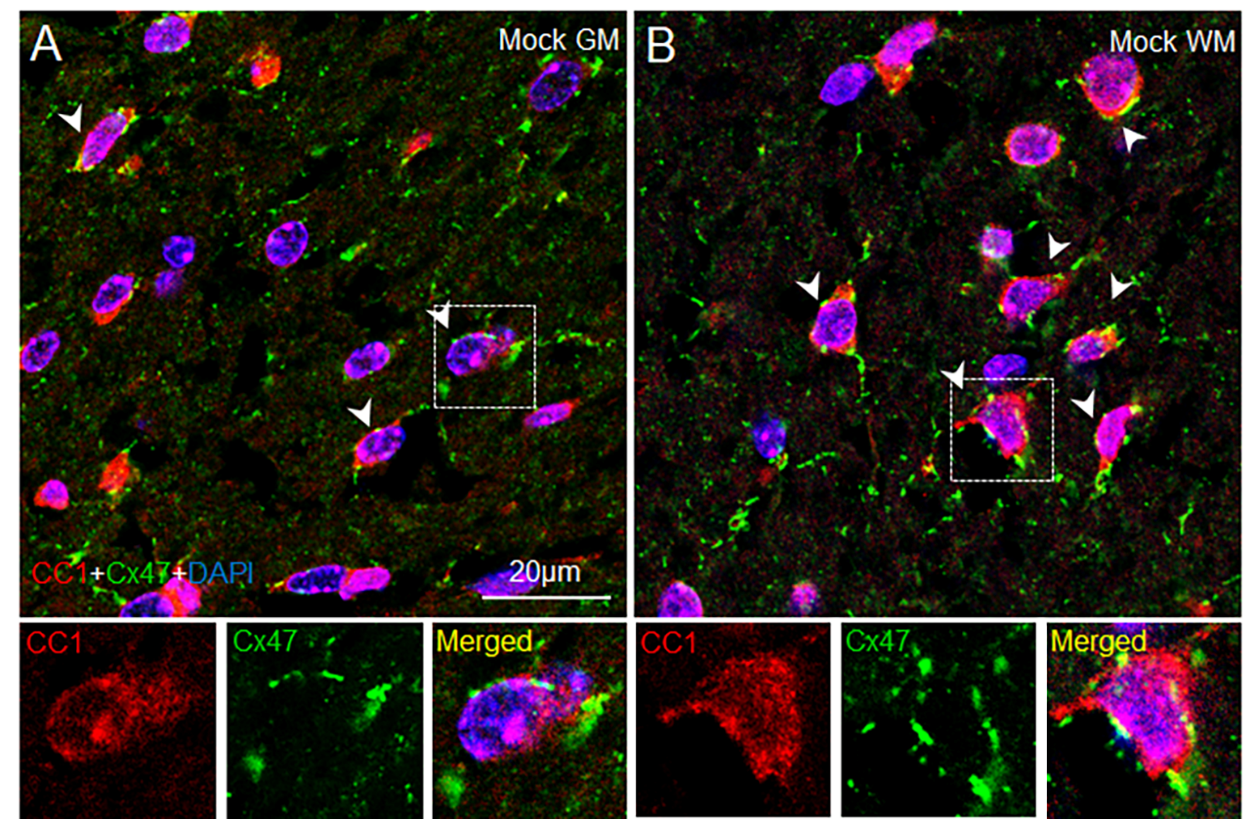
**

**Fig. S3** Cx47 GJ expression in CC1-labelled mature oligodendrocytes of thoracic spinal cord sections of mock-infected control mice at 30-days post-infection (pi). (A, B) Representative confocal images from grey matter (GM, A) and white matter (WM, B) areas of thoracic spinal cord sections double immunostained for anti-Cx47 and anti-CC1 antibodies. Mock infected spinal cord show Cx47 GJs mostly in the form of a dense ring outlining the periphery of CC1-labelled mature OLs in the GM (A) and WM (B) areas.

**
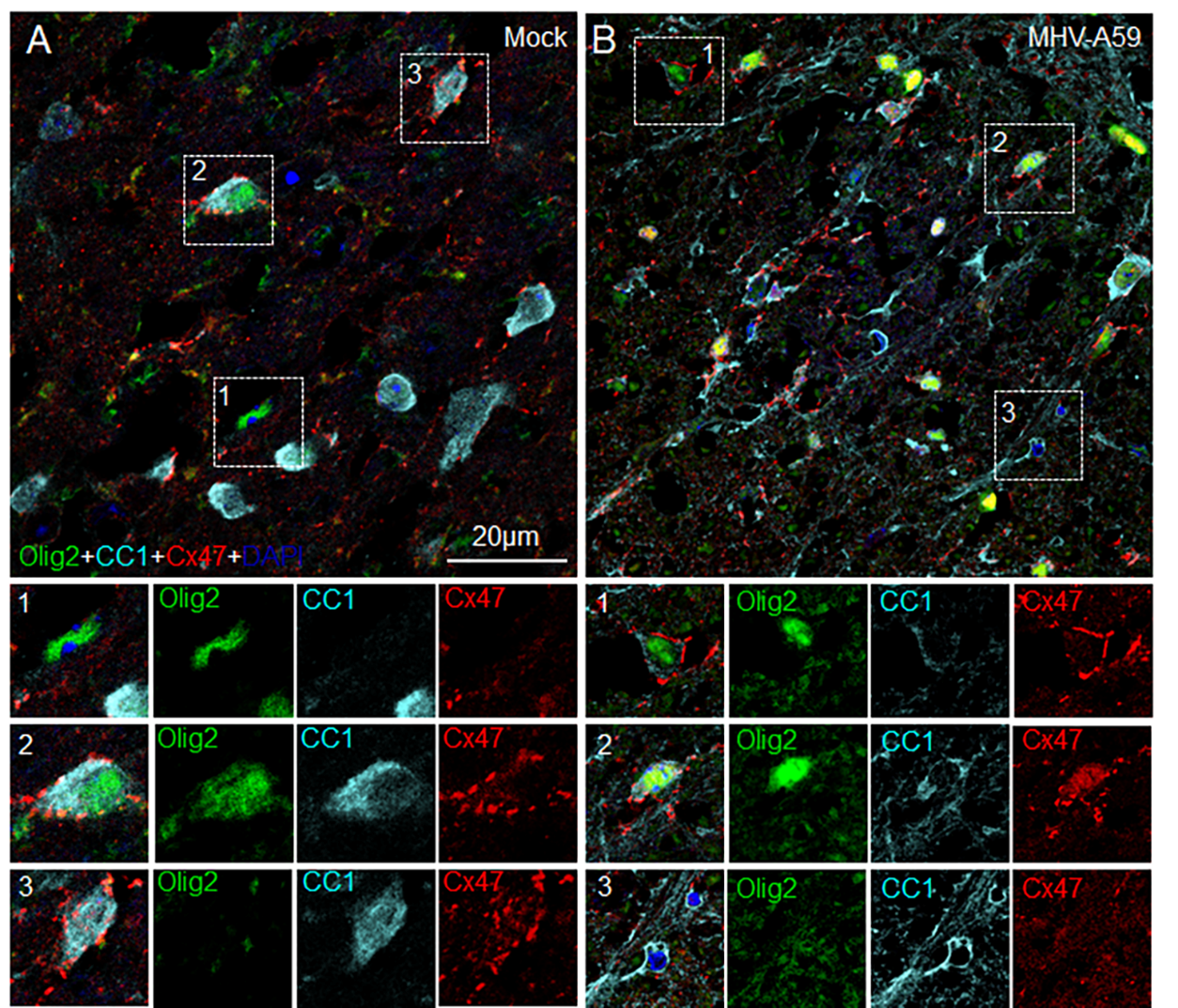
**

**Fig. S4** Cx47 GJ expression in Olig2+/CC1-negative oligodendrocyte precursor cells in spinal cord sections following MHV-A59 infection at 30-days post-infection (pi). (A, B) Representative confocal images of spinal cord white matter area triple immunostained for anti-Cx47, anti-Olig2 and anti-CC1 antibodies. Note that Olig2-positive and CC1-negative (Olig2+/CC1-) precursor cells exhibit Cx47 GJ puncta in MHV-A59 infected spinal cord (B, inset 1) but not in mock-infected controls (A, inset 1). In contrast, CC1-labelled mature OL populations (Olig2+/CC1+ and Olig2-/CC1+) which show prominent Cx47 GJ expression in control (A, insets 2 and 3) tissue exhibit a significant loss or diffused staining of Cx47 in infected sections (B, insets 2 and 3).

**
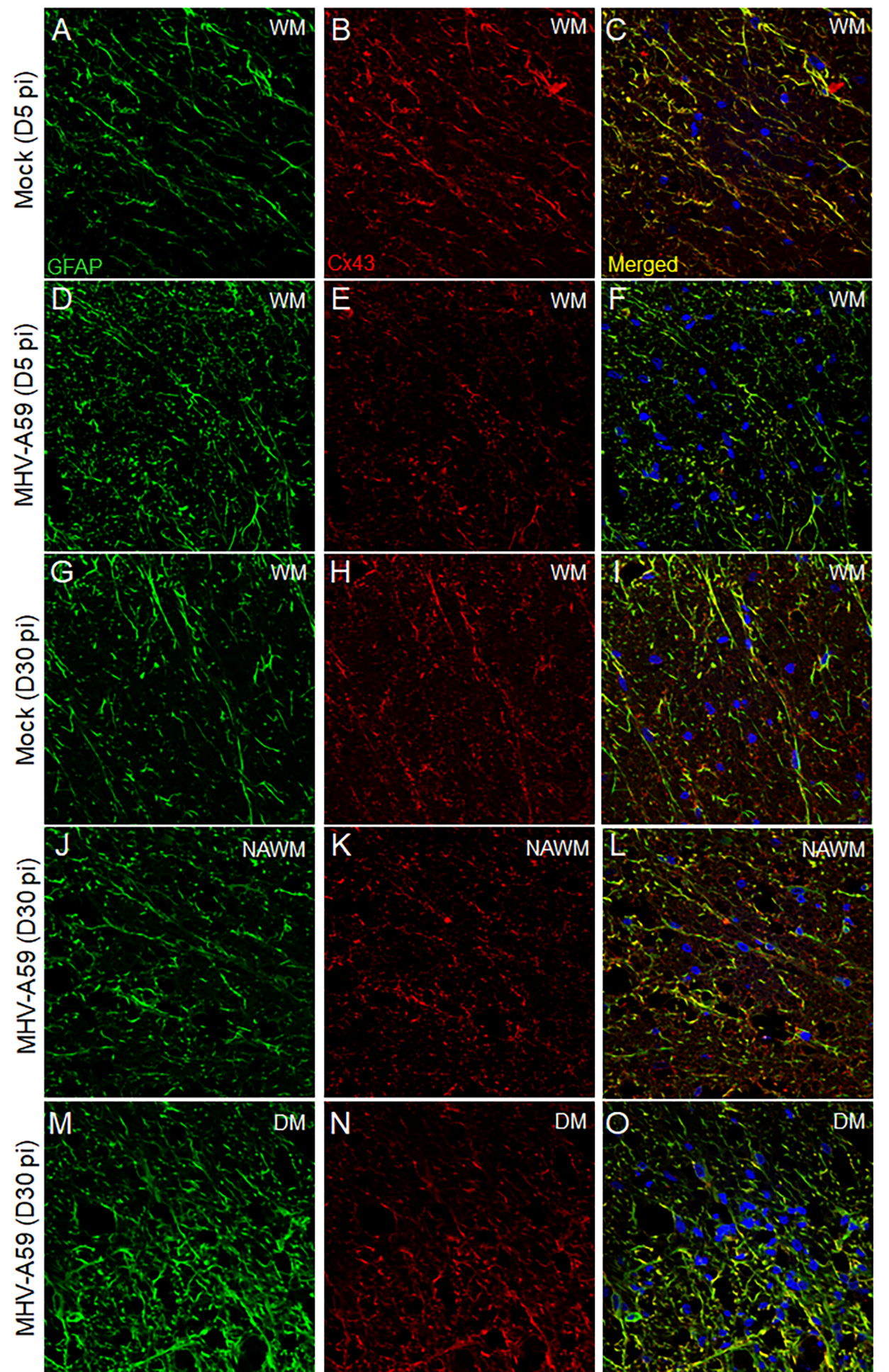
**

**Fig. S5** Alterations in Cx43 expression in association with altered GFAP levels in mock and MHV-A59 infected spinal cord. (A-F) Representative confocal images of thoracic white matter (WM) areas of mock (A-C) and MHV-A59 infected (D-F) mice during the acute infection stage (5 days pi). Note the upregulation in GFAP staining intensity and downregulation of Cx43 punctate staining in in the infected tissue (D-F) compared to control (A-C) at 5 days pi. (G-O) Representative confocal images of thoracic white matter (WM) areas of mock (G-I) and MHV-A59 infected (J-O) mice during the chronic demyelinating stage (30 days pi). The normal appearing white matter (NAWM) region of infected thoracic cord sections (J-L) show GFAP and Cx43 labelling similar to WM region of respective control (G-I). The demyelination lesions (DM) exhibit increased GFAP staining (M-O) compared to control WM (G-I) while Cx43 levels appear to be restored back to levels similar to control by 30 days pi.


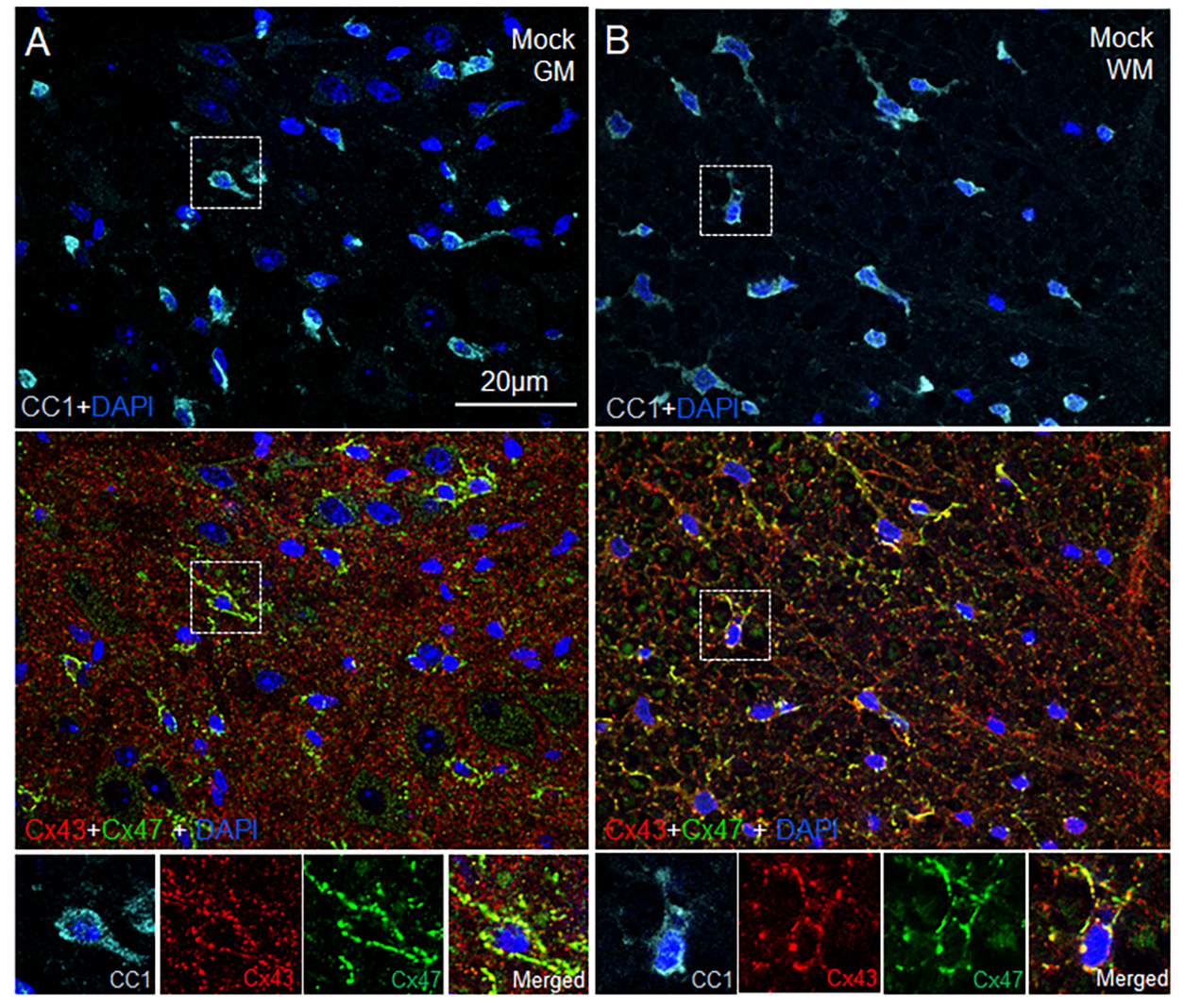


**Fig. S6** Cx43-Cx47 GJ expression in thoracic spinal sections from mock-infected control mice at 5-days post-infection. (A, B) Representative confocal images of grey (GM, A) and white matter (WM, B) areas of mock-infected thoracic spinal cord sections at 5-days post-infection triple immunostained for anti-Cx47, anti-Cx43 and anti-CC1 antibodies. Cx43-Cx47 GJs typically outline the periphery of CC1-labelled mature OLs (insets) in control spinal cord sections.

**
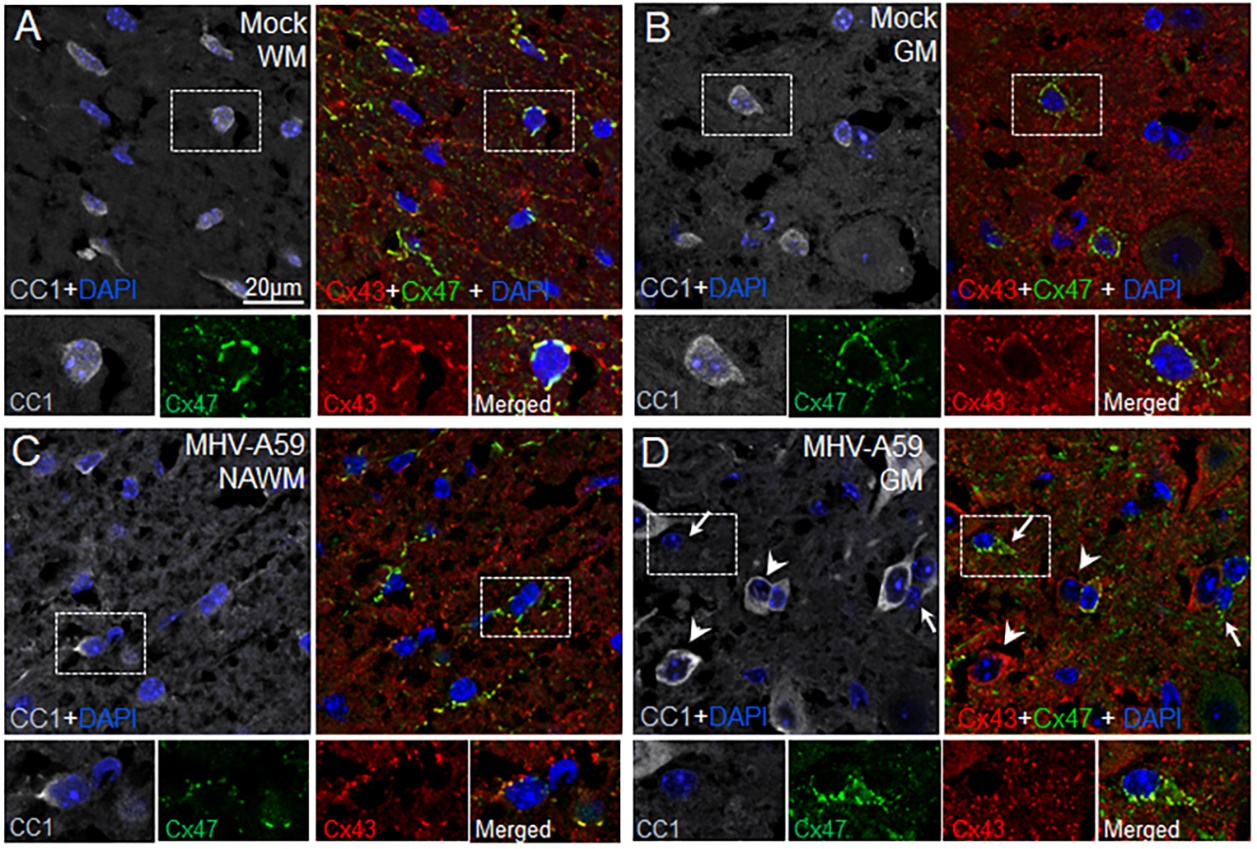
**

**Fig. S7** Altered Cx43-Cx47 astrocyte-oligodendrocyte GJ expression upon MHV-A59 infection at chronic demyelination in cervical spinal cord. (A-D) Representative confocal images from mock (A and B) and virus-infected spinal cord sections (C and D) at 30-days post-infection (pi) triple immunostained for anti-Cx47, anti-Cx43 and anti-CC1 antibodies. Note that loss of Cx43-Cx47 GJ coupling in CC1-labelled oligodendrocytes in normal appearing white (WM, C) and grey (GM, D) matter areas upon viral infection compared to control sections (A, B). Arrows indicate appearance of Cx47 GJ puncta in CC1-negative cells in infected tissue (D and inset), while arrowhead depicts loss of Cx47 GJs in CC1-labelled cells.

**
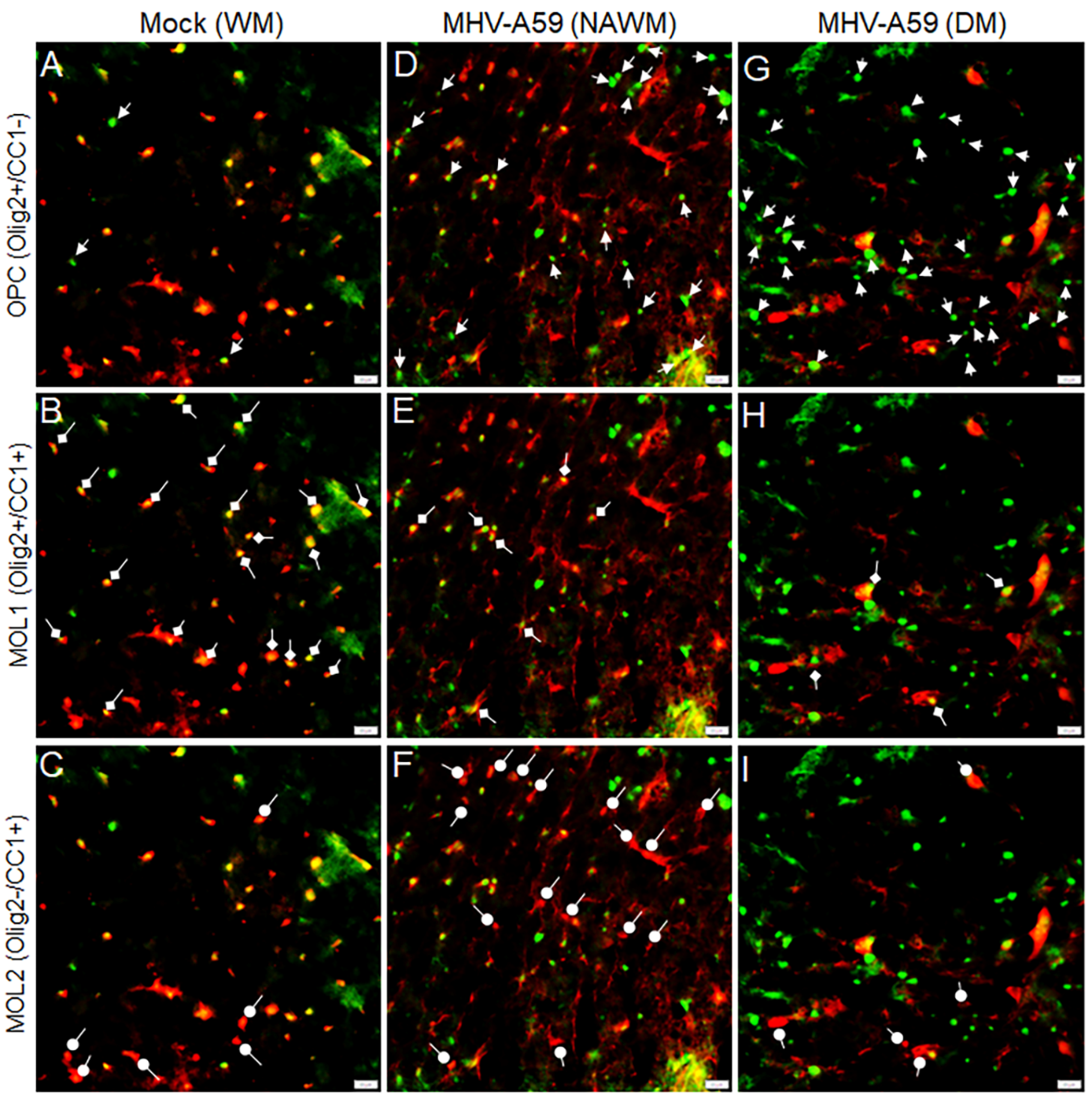
**

**Fig. S8** Representative photomicrographs illustrating alterations in the proportions of the oligodendrocyte (OL) lineage cell populations in the MHV-A59 infected thoracic spinal cord tissue at 30 days pi. (A-C) Representative photomicrograph of a single image field from the spinal cord white matter (WM) of a mock-infected control mouse showing the proportion of Olig2+/CC1- OPCs (A, open arrows), Olig2+/CC1+ MOL1 (B, diamond arrows) and Olig2-/CC1+ MOL2 (C, circle arrows) populations. (D-F) Representative photomicrograph of a single image field from the spinal cord normal appearing white matter (NAWM) of an MHV-A59 infected mouse showing the proportion of Olig2+/CC1- OPCs (D, open arrows), Olig2+/CC1+ MOL1 (E, diamond arrows) and Olig2-/CC1+ MOL2 (F, circle arrows) populations. (G-I) Representative photomicrograph of a single image field from a demyelinated (DM) region the spinal cord of an MHV-A59 infected mouse showing the proportion of Olig2+/CC1- OPCs (G, open arrows), Olig2+/CC1+ MOL1 (H, diamond arrows) and Olig2-/CC1+ MOL2 (I, circle arrows) populations. Olig2 staining is shown in green while CC1 labelling is in red. Scale bar, 20μm.

**
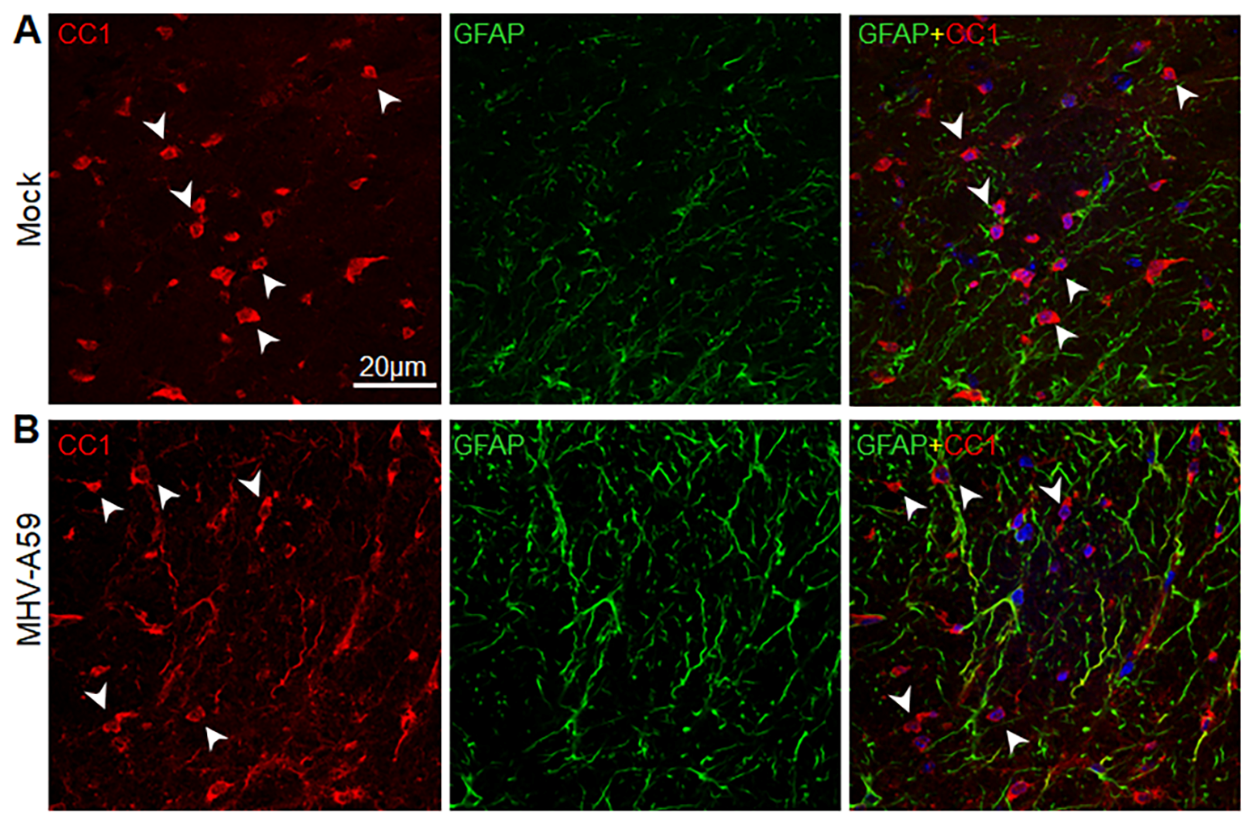
**

**Fig. S9** Representative confocal images showing that CC1-labelled cells lack astrocytic identity. (A, B) Double immunofluorescence labelling of spinal cord sections from mock (A) and MHV-A59 (B) infected mice with for oligodendrocyte marker CC1 and astrocyte marker GFAP. Note that CC1-labelled cells (arrows) lack GFAP labelling in both mock-infected control and virus infected sections.
